# Generic self-stabilization mechanism for biomolecular adhesions under load

**DOI:** 10.1038/s41467-022-29823-2

**Published:** 2022-04-22

**Authors:** Andrea Braeutigam, Ahmet Nihat Simsek, Gerhard Gompper, Benedikt Sabass

**Affiliations:** 1 Theoretical Physics of Living Matter, Institute for Biological Information Processes, Forschungszentrum Jülich, 52425 Jülich, Germany; 2 Department of Veterinary Sciences, Institute for Infectious Diseases and Zoonoses, Ludwig-Maximilians-Universität München, 80752 Munich, Germany

**Keywords:** Cellular motility, Focal adhesion, Biological physics, Statistical physics

## Abstract

Mechanical loading generally weakens adhesive structures and eventually leads to their rupture. However, biological systems can adapt to loads by strengthening adhesions, which is essential for maintaining the integrity of tissue and whole organisms. Inspired by cellular focal adhesions, we suggest here a generic, molecular mechanism that allows adhesion systems to harness applied loads for self-stabilization through adhesion growth. The mechanism is based on conformation changes of adhesion molecules that are dynamically exchanged with a reservoir. Tangential loading drives the occupation of some states out of equilibrium, which, for thermodynamic reasons, leads to association of further molecules with the cluster. Self-stabilization robustly increases adhesion lifetimes in broad parameter ranges. Unlike for catch-bonds, bond rupture rates can increase monotonically with force. The self-stabilization principle can be realized in many ways in complex adhesion-state networks; we show how it naturally occurs in cellular adhesions involving the adaptor proteins talin and vinculin.

## Abstract (web summary)

Cellular adhesions have the remarkable property that they adapt their stability to the applied mechanical load. Here, authors describe a generic physical mechanism that explains self-stabilization of idealized adhesion systems under shear.

## Introduction

Multicellular organisms are held together by complex biomolecular adhesion structures. For decades, cellular adhesions have been extensively studied, both, as a paradigm for fundamental biophysical mechanisms, as well as to understand their essential biological function. Yet, a very fundamental property of cell–matrix adhesions remains mysterious—their ability to adapt their size to the mechanical load. How is it possible—from a physics perspective—that the strength of adhesions increases under load?

Cell–matrix adhesions, also called focal adhesions, are crucial for cell physiology^1,2^, cell motility^3^, cancer metastasis^4,5^, and development^6,7^. The structures consist of transmembrane integrins and adaptor proteins that connect the force-generating actomyosin cytoskeleton with the extracellular matrix. Accordingly, focal adhesions have been likened to a “molecular clutch”^8–13^. In a pioneering experiment by Riveline et al.^14^, it was shown that local application of centripetal forces to adherent cells induces focal adhesion growth^14^. Moreover, the size of focal adhesions is proportional to the load^15^. The regulatory network associated with focal adhesions is complex and several biological processes have been proposed to play a role for adhesion stability. These include mechanosensitive activation of integrins^11^, catch-bond behavior of integrins and vinculin-F-actin binding^16^, non-linear mechanical response of unfolded proteins, and downstream signaling, e.g., mediated by the adaptor protein p130Cas^17,18^. During the past years, the pivotal role of the adaptor protein talin for adhesion maturation has also been established^19,20^. Talin directly transmits forces by binding with its globular head domain to integrin, while its rod domain links to F-actin^21^. Under stretch, conformation changes in talin occur, leading to an unfolding of protein domains and to the exposure of cryptic binding sites for vinculin^22–24^. Vinculin, in turn, further recruits F-actin and thereby strengthens the linkage^25–27^. Moreover, other adhesion types such as adherens junctions are also capable of a load adaptation based on unfolding and recruitment of further molecules^28^. In spite of the considerable amount of theoretical approaches^29–37^ and pioneering work combining modeling and experiment^12,38,39^, the physical principle underlying load adaptation of focal adhesions remains largely not understood.

We propose a generic mechanism through which molecular adhesions can harness mechanical load for adapting their size and stability without active feedback. A minimal model is employed, which relies on a combination of unfolding of adhesion molecules under force^39,40^, the dynamical exchange of molecules with a reservoir^29,30^, and possibly the recruitment of additional molecules that stabilize the unfolded conformations, all under the constraint of thermodynamical consistency. Under tangential stress, the state occupations shift, leading to a growth of the adhesion under increasing load, until the adhesion ultimately fails at very high loads. This mechanism of adaptation is simple and robust. Numerical analysis shows that the key property allowing adaptation of our molecular state-networks is that some states are separated from the reservoir. We show how the mechanism is naturally realized by the talin-vinculin system at focal adhesions and perhaps in other bioadhesions. Moreover, the proposed mechanism is general enough that it could possibly be implemented in engineered biomimetic systems.

## Results

### Molecular adhesion model

**Fig. 1:**
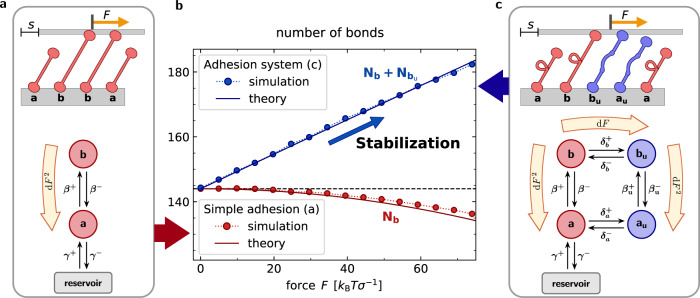
Adhesion self-stabilization. **a** Illustration of the basic adhesion model which consists of unbound molecules *a* and bonds *b* that connect two parallel rigid planes. A tangential load stretches all molecules in state *b* as it shifts the upper boundary by *s*. Molecules can transition between states *b* and *a* with extension-dependent rupture and binding rates given by *β*^−^(*h*) and *β*^+^(*h*). Molecules can enter or leave the adhesion cluster with rate constants given by *γ*^±^﻿. The decrease of the ratio of bound to unbound molecules under a small force d*F* is a second order effect (orange arrow). **b** Mean number of bonds in steady state as a function of loading force. Markers show simulation results and lines correspond to approximate analytical results. In the basic model, an increased load on the adhesion reduces the number of bonds (red). In contrast, an increased load produces a growth of the number of bonds in the generalized model (blue). **c** Illustration of the generalized adhesion model incorporating molecule unfolding and refolding with rates ${\delta }_{a,b}^{\pm }(h)$ as well as a molecule-exchange with the reservoir. Mechanical load drives the system out of equilibrium, shifting the state occupations, as indicated by orange arrows for changes up to second order. See also Supplementary Movies 1, 2.

We consider a generic adhesion system consisting of molecules that form harmonic bonds between two planar, parallel surfaces, see Fig. 1. The stretch of each molecule, i.e., the difference between its actual length and rest length, is denoted by *h* and the spring constant is *κ*. The bottom surface is fixed in space and a constant tangential loading force *F* is exerted on the top, leading to a time-dependent tangential shift *s*. The model is two-dimensional and forces normal to the surface are not considered. The mean number of molecules in the adhesion system is denoted by *N*. A molecule reservoir is assumed and the stochastic molecule exchange with the adhesion system is determined by the rate constants *γ*^+^ and *γ*^−^. Individual molecules undergo stochastic transitions between different states in the adhesion. All stochastic transition rates are chosen to fulfill detailed balance in equilibrium, i.e., when *F* = 0, which avoids unphysical energy injection that could produce an apparent motor-like behavior of the system.

In a first, basic model, see Fig. 1a, molecules from the reservoir associate reversibly with the adhesion system via the state *a*, in which they have not yet formed a bond between the upper and lower surfaces. The stretch *h* of unbound molecules in state *a* undergoes thermal equilibrium fluctuations with magnitude $\sigma =\sqrt{{k}_{{{{{{{{\rm{B}}}}}}}}}T/\kappa }$, where *k*_B_*T* is the thermal energy scale. Molecules can then form a bond between the surfaces and this state is denoted by *b*. *N*_*a*_ and *N*_*b*_ are the average numbers of molecules in states *a* and *b*, respectively. The rate of stochastic bond formation is denoted by *β*^+^(*h*) and is to be maximal when the stretch equals an optimal binding distance ∣*h*∣ = *ℓ*_b_. For molecule unbinding, we focus on slip-bond dynamics with rupture rates *β*^−^(*h*) that increase exponentially with bond stretch, see Methods 4A, B. To ensure detailed balance in equilibrium, we demand $\beta (h)={\beta }^{+}(h)/{\beta }^{-}(h)=\exp (-{{{{{{{{\mathcal{E}}}}}}}}}_{\beta }/{k}_{{{{{{{{\rm{B}}}}}}}}}T)$, where ${{{{{{{{\mathcal{E}}}}}}}}}_{\beta }$ is the free energy difference between the unbound and bound molecule.

In a second, generalized model, see Fig. 1c, the molecules can undergo sudden conformational changes leading to molecule unfolding. Unfolded states are denoted by a subscript *u*. The unbound, unfolded state is *a*_u_ and the average number of molecules in this state is ${N}_{{a}_{{{{{{{{\rm{u}}}}}}}}}}$. The bound, unfolded state is *b*_u_ with average number of molecules ${N}_{{b}_{{{{{{{{\rm{u}}}}}}}}}}$. Unfolding is modeled as a thermally assisted escape over a single energy barrier. To satisfy detailed balance at equilibrium, the ratio of forward and reverse rate is given by a Boltzmann factor involving the free energy difference. Details are given in Methods 4C. We assume that mechanical relaxations occur instantaneously and viscous damping is neglected, so that the sum of the forces borne by the bonds equals the applied load *F* at all times. Stochastic bond dynamics is simulated with an exact algorithm, see Supplementary Notes II. In the following, the mean number of bonds formed by all molecules, irrespective of their unfolding state, is denoted by *N*_*B*_. *N*_*A*_ is the corresponding mean overall number of unbound molecules.

### Self-stabilization of macromolecular adhesions

We first consider the basic adhesion model, in which molecules do not change conformation, see Fig. 1a and Supplementary Movie 1. Simulations reveal that a load *F* can lead to a quasi-stationary adhesive state, where perpetual rupture and binding events result in a stationary state of persistent tangential sliding of the surfaces. For these adhesions, the mean number *N*_*B*_ of bonds always decreases monotonically with *F*, see Fig. 1b. Therefore, increasing load on adhesions consisting of simple molecules promotes adhesion failure characterized by rupture of all bonds. Next, we consider the generalized adhesion model consisting of molecules that can undergo an unfolding transition under force, see Fig. 1c and Supplementary Movie 2. For simplicity, we assume that unfolding only entails an increase in the rest length, while the elastic properties remain unchanged. Remarkably, the average number of bonds *N*_*B*_ now initially grows with increasing load *F*, see Fig. 1b, which prevents early adhesion failure. This striking effect, which we call “self-stabilization”, is the central finding of this work. We emphasize that this effect crucially depends on the exchange of “folded” molecules (state *a*) with the reservoir, so that the transitions *a* → *b* → *b*_u_ lead to a depletion of *a* molecules, which can be replenished from the reservoir, thereby leading to an increase of the adhesion size. This mechanism is explained in detail below.

The simulation results can be corroborated with an analytical mean-field approximation^33^. The number distributions of molecules with stretch *h* are denoted by *n*_*b*_(*h*) and *n*_*a*_(*h*), for the bound and unbound states, respectively. For the basic adhesion model without molecule unfolding, a drift-reaction equation is assumed where the average sliding velocity of the adhesion $v=\langle \dot{s}\rangle$ stretches the molecules, so that

1 $${\partial }_{t}{n}_{b}(h)+v{\partial }_{h}{n}_{b}(h)={\beta }^{+}(h){n}_{a}(h)-{\beta }^{-}(h){n}_{b}(h).$$

Only stationary solutions with ∂_*t*_*n*_*b*_(*h*) = 0 are considered. The average total number of molecules in the adhesion is obtained as $N={N}_{B}+{N}_{A}=\int\nolimits_{-\infty }^{\infty }\,[{n}_{b}(h)+{n}_{a}(h)]{{{{{{{\rm{d}}}}}}}}h$, where the stretch of the unbound molecules obeys a Gaussian distribution, ${n}_{a}(h)\propto {{{{{{{\mathcal{N}}}}}}}}(0,{\sigma }_{a}^{2})$. The non-linear equations are solved by expanding the distributions for small absolute values of $\tilde{v}=v/({k}_{\beta }{\sigma }_{b})$, where the binding constant *k*_*β*_ is employed as time unit and the stretch standard deviation of bound molecules *σ*_*b*_ as length unit. We expand, e.g., ${n}_{b}(h)={n}_{b}^{* }(h)+\tilde{v}{n}_{{b}_{1}}(h)+\frac{1}{2}{\tilde{v}}^{2}{n}_{{b}_{2}}(h)+{{{{{{{\mathcal{O}}}}}}}}({\tilde{v}}^{3})$. Here and in the following, asterisks (^*^) denote equilibrium quantities calculated with *F* = 0 and tildes $\tilde {()}$ are used to denote non-dimensionalized quantities. Using the additional assumption that the optimal molecule stretch for binding, *ℓ*_b_, is much smaller than the typical length fluctuations, ${\tilde{\ell }}_{{{{{{{{\rm{b}}}}}}}}}={\ell }_{{{{{{{{\rm{b}}}}}}}}}/{\sigma }_{b}\ll 1$, we find

2 $${N}_{B}-{N}_{B}^{* }\approx -{(2/\pi )}^{1/2}{\tilde{\ell }}_{{{{{{{{\rm{b}}}}}}}}}{N}_{B}^{* }\,{\tilde{v}}^{2}\propto -{F}^{2},$$

where, due to symmetry under reversal of the force direction, to leading order $\tilde{v}\propto \,F$. For the general case, where *ℓ*_b_ ≪ *σ*_*b*_ does not hold, the first non-vanishing correction to the equilibrium solution for the bonds *N*_*B*_ can be shown to still be of the order ${\tilde{v}}^{2}$ and strictly negative, see Supplementary Notes III. Thus, tangential force reduces the number of bonds and thereby always destabilizes simple adhesions consisting of molecules that do not undergo conformation changes, as expected intuitively.

To support the effect of self-stabilization in the generalized model shown in Fig. 1c with analytical theory, we supplement Eq. (1) by two additional equations for binding and unfolding transitions, see Supplementary Notes IV. For $| \tilde{v}| \ll 1$, the overall number of bound molecules, ${N}_{B}={N}_{b}+{N}_{{b}_{{{{{{{{\rm{u}}}}}}}}}}$, can be expanded as

3 $${N}_{B}-{N}_{B}^{* }\approx {N}_{B,1}\tilde{v}+{N}_{B,2}\,{\tilde{v}}^{2}/2\propto | F| .$$

The leading contribution is linear in $\tilde{v}$ with a positive coefficient *N*_*B*,1_ (obtained from numerical analysis), as required for self-stabilization! The second-order coefficient *N*_*B*,2_ can be positive or negative, see Supplementary Fig. 3. Hence, analytical models confirm the existence of a self-stabilization regime where the number of adhesion bonds initially increases with load.

### Mechanism of self-stabilization

**Fig. 2:**
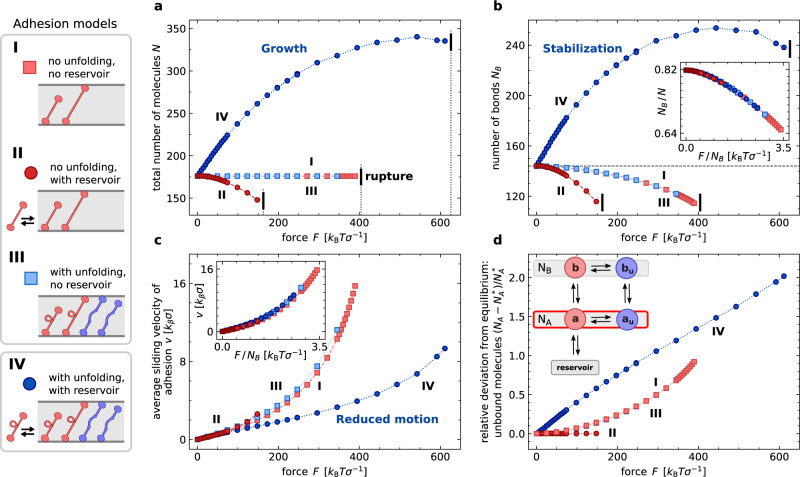
Comparison of models I–IV for adhesions with and without molecule unfolding and reservoir exchange. Steady-state quantities are plotted until forces at which first complete adhesion ruptures occur. **a** Averaged total number of molecules *N*. **b** Averaged overall number of bond *N*_*B*_ formed by folded and unfolded molecules in steady state. Only model IV shows self-stabilization where the mean number of bonds increases initially with force. **c** Continuous rupture-rebinding transitions lead to a relative motion of the two planes bounding the adhesion. Self-stabilization reduces the motion. **d** Relative deviation of the average number of unbound molecules *N*_*A*_ from equilibrium. Note the increased molecule accumulation in the unbound states for self-stabilization. See Supplementary Notes I for parameters.

To obtain deeper insight into the necessary ingredients for self-stabilization, and to demonstrate the essential contribution of the reservoir, we compare several variants of the basic models, see Fig. 2—such as a simple adhesion model with fixed molecule number (model I), a model comprising a molecule reservoir and therefore adhesions of variable size (model II), a model with a fixed number of molecules that can unfold under force (model III), and a model combining unfolding molecules with a molecule reservoir (model IV). Some results from models II and IV are also shown in Fig. 1. For models I–III, the mean number of bonds *N*_*B*_ decreases with force, see Fig. 2b. For model II with variable system size, even the number of molecules in the adhesion decreases with force, leading to an earlier adhesion failure on average. Thus, neither a variable adhesion size nor molecule unfolding alone result in self-stabilization. In model IV, which combines a variable adhesion size with molecule unfolding, both the total number of molecules *N* and the number of bonds *N*_*B*_ initially grow with increasing load on the adhesion, Fig. 2a, b. The increased number of bonds improves load sharing among the molecules. One consequence is a significant reduction of the sliding motion of the adhesion, Fig. 2c. The inset in Fig. 2b shows that the bound fractions of molecules, *N*_*B*_/*N*, as a function of *F* collapse onto a single master curve for all models (I–IV). Hence, self-stabilization results from force-induced growth of the adhesion and not from changes of the rupture properties of individual molecules. This is the key difference to established catch-bond models, where individual molecules exhibit an increase of bond lifetime within limited force regimes.

Figure 2d illustrates the underlying mechanism of self-stabilization. The tangential load *F* causes a shift of state occupations. The load increases the occupations of state *b*_u_ and *a*_u_, compare Supplementary Notes IV. Meanwhile, the state *a*, representing unbound, folded molecules, is in contact with the reservoir and molecules are replenished here, which allows a concurrent increase of the overall molecule number. It is important to note that without the reservoir, self-stabilization cannot occur because it requires growth of the adhesion cluster. The recruitment of molecules from the reservoir crucially depends on the intermediate state *a*_u_* not* to be in contact with the reservoir. For systems where both unbound states *a* and *a*_u_ are connected to the reservoir, we find numerically that the self-stabilizing first-order correction *N*_*B*,1_ is two orders of magnitude smaller than the second order correction *N*_*B*,2_, see Supplementary Fig. 12. In contrast, if only state *a* is connected to the reservoir as in model IV, *N*_*B*,1_ and *N*_*B*,2_ are of the same order order of magnitude, which enables self-stabilization, see Supplementary Fig 3.

**Fig. 3:**
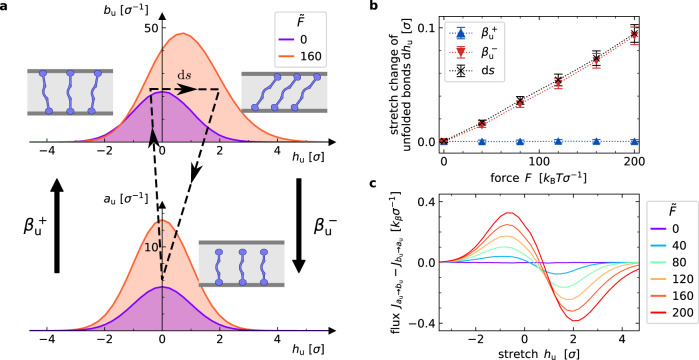
Constant mechanical load maintains a non-equilibrium steady state. **a** Stretch distributions for unfolded-molecule states *b*_u_(*h*_u_) and *a*_u_(*h*_u_). Binding occurs symmetrically around *h*_u_ = 0. Bonds exhibit a positively skewed stretch distribution under non-zero force. Cyclic stretch changes occur through binding, loading, and rupture (dashed line). **b** Average stretch changes during binding (${\beta }_{{{{{{{{\rm{u}}}}}}}}}^{+}$), rupture (${\beta }_{{{{{{{{\rm{u}}}}}}}}}^{-}$), and the average acquired stretch in the bound state d*s* for unfolded molecules. Error bars indicate sample standard deviation. c) Stretch-dependent flux-balance distributions for binding and unbinding of unfolded molecules. At vanishing forces, fluxes are balanced for all *h*_u_. For *F* > 0, a net probability flux into state *b*_u_ at negative stretches and a net flux out of state *b*_u_ at positive stretches is found. Fluxes are obtained by binning the transition rates that are chosen in steady state simulation trajectories with a bin width 0.25*σ*. Parameter values are given in Supplementary Notes I.

In general, load-dependent state-occupation statistics like those implied in our models do not always require non-equilibrium conditions. For the presented system, however, the load *F* invariably drives the systems out of equilibrium by inducing continuous fluxes. For illustration, consider the binding and rupture transition of unfolded molecules in Fig. 3. While the distribution of molecule stretches *h*_u_ is symmetric for unbound molecules, bound molecules have a skewed stretch distribution due to the force *F* > 0, see Fig. 3a. Binding and rupture effectively cause circular fluxes in stretch-state space, illustrated by the dashed lines in Fig. 3a. Figure 3b shows that the average stretch change per rupture event only vanishes for *F* = 0 and increases with *F* > 0. The resulting net occupation-probability flux from *a*_u_ to *b*_u_ is shown in Fig. 3c. Note that the mechanical loading can also drive other occupation-probability fluxes, depending on the network topology. Supplementary Fig. 4 shows that global balance conditions are broken for model IV when *F* > 0.

### Self-stabilization is a robust mechanism

**Fig. 4:**
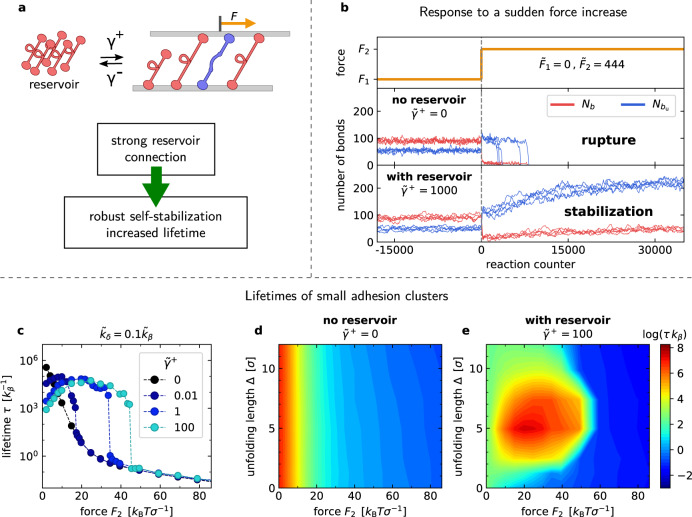
Rupture behavior and lifetimes of adhesion clusters. **a** The reservoir-exchange rates *γ*^±^ control the association and dissociation of molecules with the adhesion. **b** Exemplary force response of adhesion clusters without reservoir connection and with strong reservoir connection. A force jump amplifies molecule unfolding. Without self-stabilization, the cluster does not reach a non-equilibrium steady state but dissociates shortly after force application. **c** Average lifetimes of the adhesion clusters with N^*^ ≈ 10 for different values of *γ*^±^ with *γ* = *γ*^+^/*γ*^−^ held constant. A strong reservoir connection results in an adhesion lifetime maximum at finite, non-vanishing external forces. Average cluster lifetimes as a function of force and the unfolding length Δ for *k*_*δ*_ = 0.1*k*_*β*_ = 1/*t*_0_ and *γ*^±^ = 0 (**d**) and *γ*^±^ = 100/*t*_0_ (**e**). See Supplementary Notes I for other parameter values.

To be an efficient and universally applicable mechanism, self-stabilization must be able to compensate load changes in non-stationary conditions and should not depend on a fine-tuning of parameters. To investigate these aspects, we consider step-like load changes for different model parameters. After a load jump from *F*_1_ = 0 to *F*_2_ = *F*, adhesion clusters either dissociate quickly or reach a new non-equilibrium steady state. Self-stabilization after a load jump depends on the strength of the molecule exchange with the reservoir, which is controlled by the values of *γ*^+^ and *γ*^−^, Fig. 4a. Exemplary trajectories for the number of bonds in the folded and unfolded states, *b* and *b*_u_, are shown in Fig. 4b. The most-likely rupture forces are higher in self-stabilizing than in non-self-stabilizing adhesions, and grow with increasing reservoir-exchange rates (*γ*^±^ ≥ *k*_*β*_), see Supplementary Fig. 5, because adhesion molecules can quickly be recruited from or released into the reservoir. The self-stabilization mechanism can thus also work under dynamic load conditions.

To measure adhesion lifetimes, we simulate systems consisting of few molecules, because lifetimes increase rapidly with the number of molecules. Load-jump simulations are carried out for a reservoir-exchange rate ratio *γ* = 1 that leads to adhesions with N^*^ ≈ 10 molecules in equilibrium when *F* = 0. Lifetime is measured as the time from the force jump, *F*_1_ → *F*_2_, to the rupture of the last adhesion bond. While lifetimes of adhesions with no reservoir coupling decrease monotonically with force, i.e., show a pure slip-bond behavior, lifetimes of self-stabilizing adhesions exhibit a maximum at non-vanishing forces, see Fig. 4c. This lifetime maximum becomes more pronounced for increasing *γ*^+^ and also depends on the rate-constant ratio *k*_*δ*_/*k*_*β*_, see Supplementary Fig. 6. To further assess the robustness of the adhesion-lifetime increase to parameter choices, we vary the unfolding length *Δ*, which determines the width of the energy barrier between the native and the unfolded molecule state. For adhesions without reservoir coupling, *γ*^±^ = 0, the unfolding length *Δ* does not have a large impact on the adhesion lifetime, see Fig. 4d. However, a significant increase in adhesion lifetimes is observed for a broad range of unfolding lengths *Δ* > 0 if the reservoir coupling is strong, see Fig. 4e. Self-stabilization is less effective for very small or very large values of $\tilde{{{\Delta }}}={{\Delta }}/{\sigma }_{b}$. For the extreme case of $\tilde{{{\Delta }}}=0$, the force response of folded and unfolded states become indistinguishable, and the unfolding and refolding rates reduce to constants. For very large unfolding lengths $\tilde{{{\Delta }}}$, a high energy barrier suppresses molecule unfolding. Therefore, self-stabilization occurs in our model approximately in the range $2\le \tilde{{{\Delta }}}\le 8$, compare Supplementary Fig. 3.

### Cell–matrix adhesions

**Fig. 5:**
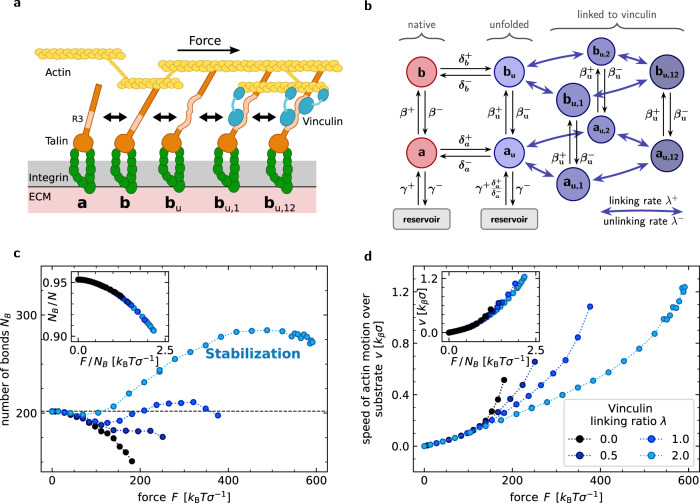
Exemplary realization of self-stabilization in focal adhesions. **a** Talin binds with its globular head to integrins and with its rod to actin filaments. The first domain to unfold under force is the R3 domain with two vinculin-binding sites. **b** State diagram for the talin-vinculin model. Averaged steady-state simulation results for different linking ratios *λ* = *λ*^+^/*λ*^−^. See Supplementary Notes I for parametrization. **c** Vinculin recruitment produces self-stabilization. **d** The relative motion of the top plane, modeling actin fibers, is reduced through vinculin-based self-stabilization.

The principle of self-stabilization can be realized in a large variety of molecular-state networks—as long as they allow for states that are increasingly populated under mechanical stress. Self-stabilization is facilitated by states that are not directly connected to the reservoir, such as *a*_u_ in the models above. As stated in the introduction, talin unfolding and its interaction with other focal-adhesion proteins have been identified as major contributors to adhesion formation. We study here a model of self-stabilization that incorporates the binding of an adaptor protein such as vinculin to talin, see Fig. 5a. The talin rod domain contains 11 cryptic vinculin binding sites. Under load, subdomains of the rod successively unfold, thereby enabling vinculin recruitment which blocks talin refolding and promotes focal-adhesion growth^41,42^. Talin unfolding typically starts at forces around 5 pN^22,25,41^ with the R3 domain, by which two vinculin binding sites are exposed. In our model, we focus on this first unfolding transition. Six additional vinculin-bound states *b*_u,_ and *a*_u,_ are introduced so that both sites in the R3 domain can be occupied independently, see Fig. 5b. Rate constants for binding and unbinding of vinculin to unfolded talin are denoted by *λ*^±^ and are assumed to be the same for all transitions. Other model parameter values are estimated according to experimental results^21,23,40^, see Supplementary Notes I.

In the vinculin-bound states, talin refolding is blocked. Thus, vinculin binding generates talin states that are not in direct contact with the reservoir. Simulation results for different values of the parameter *λ* = *λ*^+^/*λ*^−^ are shown in Fig. 5c. For *λ* = 0, all populated unbound states are connected with the reservoir, and loading cannot increase the number of molecules in these states. We observe no self-stabilization. For *λ* > 1, the number of bonds increases with force, which not only stabilizes the adhesion but also translates into a reduction of adhesion sliding, see Fig. 5d. Given the high affinity with a dissociation constant in the range [1 × 10^−7^ − 1 × 10^−8^ ] M of unfolded talin for vinculin, we expect the vinculin binding constant to be larger than unity^40,43–45^. Moreover, we conjecture that the remaining cryptic binding sites in talin that open at higher forces extend the demonstrated self-stabilization effect to larger loads. In summary, the minimal adhesion model can be applied to specific biological systems like the integrin adhesome, where vinculin binding to unfolded talin domains results in a reaction network containing molecular states that are populated by force application—and thus leads to self-stabilization. Analytical and numerical results of a simplified, corresponding mean-field model are given in Supplementary Notes VI.

## Discussion

Our theoretical model reveals a strikingly simple mechanism for a counter-intuitive load-response of adhesions, in which tangential mechanical load can result in adhesion enhancement — instead of the ubiquitous adhesion weakening and rupture. This self-stabilization relies on molecular-conformation state-networks whose occupations are changed by a mechanical load. By shifting the state occupations, the load causes a net influx of adhesion molecules from a surrounding reservoir. Notably, this self-stabilization does not essentially require extra chemical energy besides the work done by the load.

For all studied model variants, a constant, non-vanishing load results in a non-equilibrium steady state. The resulting fluxes occur in the high-dimensional state-space spanned by the continuous molecular stretch and the discrete molecular states. A first indicator for the presence of a non-equilibrium steady state is the non-vanishing average velocity with which the two adhesion planes slide relative to each other. This motion corresponds to stretch changes of bound molecules and vanishes only for the case *F* = 0 when all reactions are balanced. A second indicator for the presence of a non-equilibrium steady state is the load-dependence of the number of unbound molecules. In equilibrium, global balance imposes constant values for the average occupation numbers of unbound states. An increase of the average number of unbound molecules for *F* > 0 shows that global balance is broken and an effective flux occurs along the cycle of state transitions a$\rightleftharpoons$ b$\rightleftharpoons$ b_u_ $\rightleftharpoons$ a_u_ $\rightleftharpoons$ a, where right reaction arrows point in the direction of the net flux, compare Supplementary Fig. 4. In case that the diagram of discrete states does not contain a closed cycle, global balance conditions are fulfilled, see Supplementary Fig. 9. However, the state of the system is also defined by the stretch distributions of bound molecules not contained in the discrete diagrams. Finite load is found to always drive fluxes within the stretch distributions *b*(*h*) and *b*_u_(*h*), compare Fig. 3 and Supplementary Fig. 8.

Although this work focuses on the theoretical understanding of a generic physical mechanism, it is inspired by experimental observations on integrin-based focal adhesions, which display size adaptation to the applied load in planar cell cultures^14,15^. Different focal adhesion stabilization mechanisms presumably act in parallel, including actin polymerization, transcription regulation, integrin activation, and conformation changes of the adaptor protein talin. Contrasting to this complexity, we predict that adhesion self-stabilization emerges naturally in systems that merely incorporate an unfolding transition of adhesion molecules, like talin, and a mechanism that prevents rapid bulk-exchange of unfolded states, e.g., vinculin binding. Dissecting how different mechanisms contribute to the focal adhesion dynamics is a future challenge that is complicated by a multitude of chemically driven signaling pathways involving, e.g., the Rap1-GTP-interacting adaptor molecule^46^ or phosphorylation of vinculin or paxillin^47,48^. However, our self-stabilization model is already consistent with a number of experimental results and qualitative tests are possible: (i) Having chosen unfolding and refolding rates that correspond to measured force-dependent rates of the talin R3-domain, self-stabilization in our model requires that unfolding under stretch occurs on average before the bond ruptures, which is ensured for *Δ*_1_ > *ℓ*_b_, compare Eqs. (7) and (8). In agreement with this prediction, it has been reported that adhesion growth on rigid substrates occurs experimentally when unfolding is faster than rupture processes^23,39,41^. (ii) Our self-stabilization mechanism does not essentially depend on force transmission through secondary cross-links via adaptor proteins. In the exemplary state network analyzed above, vinculin does not bear force. This prediction is consistent with experiments showing that the vinculin head domain is sufficient to partially rescue adhesion formation^49,50^. Generally, however, self-stabilization may be improved by secondary cross-links and experimental studies with vinculin mutants can allow further assessment of specific realizations of self-stabilization. (iii) The basic condition required for our self-stabilization mechanism is that some unfolded talin states remain in the adhesion while folded talins are rapidly exchanged with a surrounding reservoir. Such conditions are consistent with talin’s large number of cryptic binding sites^20^ and the rapid exchange of talin^51^. Our model moreover predicts a break-down of self-stabilization if both exchange rates for talin are decreased simultaneously while maintaining the concentration at focal adhesions. Finally, the suggested mechanism is distinguished by requiring only a few different molecular components and no catch bonds. Thus, the occurrence of self-stabilization could also be tested in vitro under controlled conditions, e.g., with an optical-trap setup. A summary of suggestions for further comparison of the model with experiments are given in Supplementary Notes VII.

Mechanosensitive conformation changes of adhesion-linked proteins and subsequent recruitment of additional molecules are recurring motifs in many fundamental adhesion structures besides focal adhesions, for instance adherens junctions^41,52,53^ and hemidesmosomes^54^. Motor proteins also undergo mechanosensitive conformation changes and can form dynamical ensembles. Understanding the interplay of chemical kinetics and the laws of thermodynamics has greatly improved our knowledge on biomolecular systems and machines and thus the design of artificial molecular motors^55–57^. Therefore, we expect that the suggested mechanism for self-stabilization can help to decipher many physiological and pathophysiological processes controlled by mechano-chemical factors, and may even allow novel designs of bio-inspired, artificial adhesion systems.

## Methods

Since the state-transition rates depend on the molecule stretch, they depend on the force applied to the adhesion. All rates must obey the principle of microscopic reversibility. In equilibrium, the ratio of forward and reverse rates should depend only on the free energy difference ${{{{{{{\mathcal{E}}}}}}}}$ through the Boltzmann factor $\exp (-{{{{{{{\mathcal{E}}}}}}}}/{k}_{{{{{{{{\rm{B}}}}}}}}}T)$.

### Binding

Different states are allowed to have their own spring constants and corresponding quantities are denoted by subscripts, e.g., *κ*_*a*_ and ${\sigma }_{a}=\sqrt{{k}_{{{{{{{{\rm{B}}}}}}}}}T/{\kappa }_{a}}$. If not mentioned otherwise, we assume *σ*_*a*_ = *σ*_*b*_ = *σ*. For the unbound state *a*, the distribution of molecule numbers at different stretch *n*_*a*_(*h*) must be Gaussian. Using the average molecule number *N*_*a*_, the normalized stretch distribution is

4 $${n}_{a}(h)/{N}_{a}={{{{{{{{\rm{e}}}}}}}}}^{-{h}^{2}/(2{\sigma }_{a}^{2})}/\sqrt{2\pi }{\sigma }_{a}.$$

The binding rate *β*^+^(*h*) depends on the molecule stretch. Assuming a length scale *ℓ*_b_ at which the overall binding frequency *β*^+^(*h*)*n*_*a*_(*h*) is at its maximum, we employ as binding rate

5 $${\beta }^{+}(h)={k}_{\beta }{{{{{{{{\rm{e}}}}}}}}}^{-\left[{(| h| -{\ell }_{{{{{{{{\rm{b}}}}}}}}})}^{2}/(2{\sigma }_{b}^{2})-{h}^{2}/(2{\sigma }_{a}^{2})\right]+{\overline{\epsilon }}_{{{{{{{{\rm{b}}}}}}}}}},$$

where *k*_*β*_ is the intrinsic binding rate constant and ${\overline{\epsilon }}_{{{{{{{{\rm{b}}}}}}}}}={\epsilon }_{{{{{{{{\rm{b}}}}}}}}}/({k}_{{{{{{{{\rm{B}}}}}}}}}T)+\ln ({\sigma }_{a}/{\sigma }_{b})$ is an effective binding affinity. Similar rate expressions have been used previously^58^. In case of unfolded molecules, the binding rate ${\beta }_{{{{{{{{\rm{u}}}}}}}}}^{+}({h}_{{{{{{{{\rm{u}}}}}}}}})$ is given by the same expression as in Eq. (5), but *h* is replaced with *h*_u_.

### Unbinding

The unbinding rate *β*^−^(*h*) follows from the expression for *β*^+^(*h*) together with the detailed-balance condition in equilibrium^59^,

6 $${\beta }^{+}(h)/{\beta }^{-}(h)={{{{{{{{\rm{e}}}}}}}}}^{-{h}^{2}/(2{\sigma }_{b}^{2})+{h}^{2}/(2{\sigma }_{a}^{2})+{\overline{\epsilon }}_{{{{{{{{\rm{b}}}}}}}}}},$$

Solving for the unbinding rate yields

7 $${\beta }^{-}(h)={k}_{\beta }{{{{{{{{\rm{e}}}}}}}}}^{(2| h| {\ell }_{{{{{{{{\rm{b}}}}}}}}}-{\ell }_{{{{{{{{\rm{b}}}}}}}}}^{2})/(2{\sigma }_{b}^{2})+\ln ({\sigma }_{b}/{\sigma }_{a})}.$$

Thus, the stretch-dependence of the unbinding rates is of the Bell-Evans form. For unfolded molecules, the unbinding rate ${\beta }_{{{{{{{{\rm{u}}}}}}}}}^{-}(h)$ is given by same expression as in Eq. (7), but *h* is replaced with *h*_u_.

### Unfolding and refolding

The unfolding and refolding reaction is modeled as the transition between two local energy minima separated by a single barrier. The distance to the barrier is denoted by *Δ*_1_ for unfolding and by *Δ*_2_ for refolding. The total unfolding length is given by *Δ* = *Δ*_1_ + *Δ*_2_. The unfolding rates for the states *a* and *b* are defined as

8 $${\delta }_{a,b}^{+}(h)={k}_{\delta }{{{{{{{{\rm{e}}}}}}}}}^{(2{{{\Delta }}}_{1}h-{{{\Delta }}}_{1}^{2})/(2{\sigma }_{a,b}^{2})-{\tilde{\epsilon }}_{{{{{{{{\rm{f}}}}}}}}}},$$

where ${\tilde{\epsilon }}_{{{{{{{{\rm{f}}}}}}}}}={\epsilon }_{{{{{{{{\rm{f}}}}}}}}}/({k}_{{{{{{{{\rm{B}}}}}}}}}T)$ is a constant energy contribution for the conformation change. The corresponding folding rates are given by

9 $${\delta }_{a,b}^{-}(h)={k}_{\delta }{{{{{{{{\rm{e}}}}}}}}}^{(-2{{{\Delta }}}_{2}h-{{{\Delta }}}_{2}^{2})/(2{\sigma }_{a,b}^{2})}.$$

The ratio of unfolding and folding rates is given by a Boltzmann factor containing the energy difference between folded molecules with stretch *h* and unfolded molecules with stretch *h* − *Δ*. For molecules in the bound state *b* the result is

10 $${\delta }_{b}^{+}(h)/{\delta }_{b}^{-}(h-{{\Delta }})={{{{{{{{\rm{e}}}}}}}}}^{(2h{{\Delta }}-{{{\Delta }}}^{2})/(2{\sigma }_{b}^{2})-{\tilde{\epsilon }}_{{{{{{{{\rm{f}}}}}}}}}}.$$

Together, the binding and folding rates fulfill Kolmogorov’s criterion for networks containing cycles^55,60^. For example, the criterion for the network shown in Fig. 1c is given by

11 $$1=\frac{{\beta }^{+}(h)\,{\delta }_{b}^{+}(h)\,{\beta }_{{{{{{{{\rm{u}}}}}}}}}^{-}(h-{{\Delta }})\,{\delta }_{a}^{-}(h-\Delta)}{{\beta }^{-}(h)\,{\delta }_{b}^{-}(h-{{\Delta }})\,{\beta }_{{{{{{{{\rm{u}}}}}}}}}^{+}(h-{{\Delta }})\,{\delta }_{a}^{+}(h)},$$

where the stretch of folded bonds *h* reduces to *h* − *Δ* due to unfolding.

## Supplementary information

Supplementary Information

Description of Additional Supplementary Files

Supplementary Movie 1

Supplementary Movie 2

## Data Availability

Source data for the presented figures are provided with this paper. Further simulation data that is generated and analyzed during the current study is available from the corresponding author on reasonable request. Source data are provided with this paper.

## Source data

Source Data

## Code availability

Code for the simulation of cluster trajectories is available from the corresponding author on reasonable request.
