# Supplementary material for: Generic self-stabilization mechanism for biomolecular adhesions under load
Source: Nat Commun. 2022 Apr 22;13:2197. doi: 10.1038/s41467-022-29823-2 (PMC9033785; doi:10.1038/s41467-022-29823-2)
Supplement: Supplementary file 1 — Supplementary Information [file 41467_2022_29823_MOESM1_ESM.pdf]

Supplementary Information: Generic self-stabilization mechanism for biomolecular adhesions under load

Andrea Braeutigam,[1] Ahmet Nihat Simsek,[1,2] Gerhard Gompper,[1] and Benedikt Sabass[1,2]

[1] *Theoretical Physics of Living Matter, Institute for Biological Information Processes, Forschungszentrum Jülich, 52425 Jülich, Germany*

[2] *Institute for Infectious Diseases and Zoonoses, Department of Veterinary Sciences, Ludwig-Maximilians-Universität München, 80752 Munich, Germany*

Supplementary Notes I. PARAMETERS

The minimal model for self-stabilization presented in the main text is motivated by focal cell-matrix adhesions. Model parameters were chosen accordingly. Supplementary Table 1 contains all model parameters and their numerical values employed for the simulations if not stated otherwise in the text.

Variable	Description	sim. model	sim. talin	unit
$k_{\mathrm{B}}T$	thermal energy	4.114	4.114	pN nm
κ_a	spring constant	0.25	0.5	$\mathrm{pN\,nm^{-1}}$
κ_b	spring constant	0.25	0.5	$\mathrm{pN\,nm^{-1}}$
σ	thermal fluctuation length	4.057	2.868	nm
k_β	rate, binding	1	1	t_0^{-1}
ℓ_{b}	binding distance	1	1	nm
ϵ_{b}	energy, binding	1.50	3.00	$k_{\mathrm{B}}T$
k_δ	rate, folding	1	100	t_0^{-1}
ϵ_{f}	energy, folding	0.50	5.83	$k_{\mathrm{B}}T$
Δ	unfolding length	10	12	nm
Δ_1	transition state distance, unfolding	5	7	nm
Δ_2	transition state distance, refolding	5	5	nm
λ^+	rate, linking	$\{0, 1, 2\}$	$\{0.0, 0.5, 1.0, 2.0\}$	t_0^{-1}
λ^-	rate, unlinking	1	1	t_0^{-1}
γ^+	rate, addition from reservoir	$\{20.0, 14.5, 11.4\}$	$\{1., 0.996, 0.991, 0.977\}$	t_0^{-1}
γ^-	rate, removal to reservoir	1	0.1	t_0^{-1}

TABLE 1. Model parameters and values employed for simulations.

In focal adhesions, mechanical force is transmitted along single or multiple proteins linked in series. In the latter case, the overall spring constant κ_b will be dominated by the element with the smallest spring constant. The stiffness of cellular adhesion proteins lies in the order of $\mathrm{pN\,nm^{-1}}$ [1, 2]. The employed value of $0.25\,\mathrm{pN\,nm^{-1}}$ is similar to values used for previous adhesion models [3–6]. The values of $\kappa_{a,b}$ used for the talin simulations were chosen to match the experimentally measured unfolding and refolding behavior, as explained below. The thermal energy scale $k_{\mathrm{B}}T$ and the spring constant κ determine the mean thermal fluctuation length σ, which is used as a length unit.

Experimentally, turnover of talin is significantly faster than turnover of integrin [7]. The binding and unbinding rates of talin are determined by the intrinsic rate k_β, the optimal binding distance ℓ_{b}, and the constant energy contribution ϵ_{b}. The intrinsic binding rate constant k_β sets the time unit t_0. The value of the binding distance ℓ_{b} agrees with the value employed in Ref. [5] and is smaller than the fluctuation length. The strength of individual bonds is determined by $F_0 = k_{\mathrm{B}}T/\ell_{\mathrm{b}} \approx 4\,\mathrm{pN}$, as the rupture rate depends on force F as $\propto \exp(F/F_0)$. The constant ϵ_{b} can be understood as an effective affinity parameter. Its value is chosen to be rather low to fix the adhesion cluster size at equilibrium, which is proportional to $\exp(\epsilon_{\mathrm{b}}/k_{\mathrm{B}}T)$, compare Refs. [8, 9].

The unfolding and refolding rates depend on k_δ, ϵ_{f}, Δ and $\Delta_{1,2}$ with $\Delta = \Delta_1 + \Delta_2$. The energy contribution ϵ_{f} determines the ratio between folded and unfolded molecules in equilibrium. It shifts the energy barrier between both states that are separated by the distances Δ_1 and Δ_2. For the simulations of talin molecules, the unfolding and refolding rate constants are chosen in accordance with the experimental results in Ref. [10]. For a time unit $t_0 = 1\,\mathrm{s}$, unfolding occurs at a rate of $0.015\,\mathrm{s^{-1}}$ at zero force. The folding and unfolding rates intersect at a force of $5\,\mathrm{pN}$, the value at which talin R3 domain unfolding and refolding is observed [10–12].

The cross-linking by additional adaptor proteins is described by making use of two rate constants $\lambda^{\pm}$. In the general simulation model, the value of these constants lies within the same order of magnitude as the intrinsic rate constants k_δ and k_β. For integrin-based adhesions, this reaction is realized by the association of vinculin to unfolded talin domains. The molecular interactions of talin and vinculin are highly complex. At low forces, vinculin binding at unfolded talin domains strengthens the adhesion [11, 13, 14]. The recruitment of vinculin is proposed to act as a negative feedback loop that stabilizes the force acting on the complex [12]. Additionally, the vinculin-talin interaction also depends on the direction of forces [15]. In our model, we assume linking-rate constants with a value comparable to the intrinsic binding rate k_β. In recent experiments addressing the association of vinculin with the talin R3 domain, vinculin binding has been observed with a rate on the order of $10^{-1}\,\mathrm{s}^{-1}$, when sufficient tension was applied to talin to induce unfolding [12].

The exchange of molecules with a reservoir is governed by the two rate constants γ^+ and γ^-. Their ratio γ is proportional to the number of molecules in the adhesion-cluster at equilibrium, i.e., for $\tilde{F} = 0$. For comparability of the results of different models, the rate for adding molecules was increased from γ^+ to $\tilde{\gamma}^+ = \gamma^+(1 + \exp(-\epsilon_\mathrm{f}/k_\mathrm{B}T))$ for the models without unfolding, and similarly when linking is included. This choice ensures the same number of molecules and bonds at $\tilde{F} = 0$. As described in the main text, the molecule-exchange with the reservoir plays a fundamental role for the self-stabilization mechanism. A strong connection to the reservoir allows the adhesion to grow with increasing force.

The construction of molecular state models for talin in integrin-based adhesions poses a challenge due to the large number of states and due to the fact that addition and removal of an adhesion molecule requires several steps *in vivo* [16, 17]. Furthermore, several pathways have been suggested for the recruitment of talin to integrin-based adhesions [18]. Values for the halftime of fluorescence recovery after photobleaching (FRAP) experiments for talin in focal adhesions are on the order of seconds or tens of seconds, depending on the cell type and substrate stiffness [19, 20]. The exchange of single molecules can therefore be expected to occur on timescales of seconds or even faster. Hence, the values for $\gamma^{\pm}$ are chosen such, that they ensure a frequent exchange of molecules with the reservoir, but still allow to observe the processes within the cluster.

Supplementary Notes II. STOCHASTIC SIMULATIONS

Molecule-state trajectories are simulated with the Gillespie algorithm [21, 22]. Its basis is provided by the stochastic formulation of chemical kinetics where the probability that a reaction i will happen in the infinitesimal time interval $\delta\tau$ is determined by the product of the available reactants or reactant pairs N_i and a parameter μ_i. Assuming $j \in [1, \ldots, m]$ available reactions, the function $P(i, \tau)$ describes the probability that the reaction i is the first one to occur after a waiting time τ in the next infinitesimal time interval. For $0 \le \tau < \infty$ we have

$$P(i,\tau) = N_i \mu_i \exp\left(-\sum_{j=1}^{m} N_j \mu_j \tau\right) . \tag{1}$$

More specifically for simulations of adhesion clusters, each bond in state b or b_u can either rupture or undergo a conformational change via stretch-dependent rates. For unbound molecules in states a and a_u, a Gaussian distribution of stretches is assumed, so that the stretch-dependence can be integrated out. This leads to four reaction channels that cover all transitions of unbound molecules. For example, the probability for one unbound molecule in state a to bind is given by $n_a\beta^+$, where the total binding rate per unbound molecule β^+ is obtained by integration of $\beta^+(h)n_a(h)/N_a$ over h,

$$\beta^+ = k_\beta \left(1 + \mathrm{Erf}\left(\tilde{\ell}_\mathrm{b}/\sqrt{2}\right)\right) \mathrm{e}^{\tilde{\epsilon}_\mathrm{b}} , \tag{2}$$

with the error function $\mathrm{Erf}(x)$. Similarly, the total unfolding and refolding rates for unbound molecules are obtained by integration of $\delta_a^{\pm}(h)n_a(h)/N_a$ as $\delta_a^+ = k_\delta \exp\left(-\tilde{\epsilon}_\mathrm{f}\right)$ and $\delta_a^- = k_\delta$. Additionally, the reservoir exchange is given by two reaction channels with constant rates γ^+ and $n_a\gamma^-$. For the adhesion cluster model without linking reactions, the sum over all reaction channel rates is therefore calculated as

$$\sum_{j=1}^{m} N_j\mu_j = n_a(\beta^+ + \delta_a^+ + \gamma^-) + n_{a_\mathrm{u}}(\beta_\mathrm{u}^+ + \delta_a^-) + \sum_{k=1}^{n_b}\Big(\beta^-(h_k) + \delta_b^+(h_k)\Big) + \sum_{k=1}^{n_{b_\mathrm{u}}}\Big(\beta_\mathrm{u}^-(h_k) + \delta_b^-(h_k)\Big) + \gamma^+ \tag{3}$$

Starting from an initial configuration, the time for the next reaction and the type of reaction are drawn according to $P(i, \tau)$ repeatedly. If a binding reaction is drawn, the initial stretch of the bond is determined according to the

distribution $\beta^+(h)n_a(h)/N_a$ or $\beta_{\mathrm{u}}^+(h)n_{a_{\mathrm{u}}}(h)/N_{a_{\mathrm{u}}}$, compare Methods A. Force balance is restored instantaneously after each event.

In response to the tangential external force, adhesion clusters either reach a quasi-steady-state or dissociate. The time and ensemble averages are calculated for trajectories that do not lead to complete dissociation during the simulation time. If not stated otherwise, 50 trajectories are tracked for $> 10^6$ single-bond transitions for the measurements of steady-state quantities, during which the tangential force is held constant. To measure adhesion lifetime, more than 200 cluster trajectories are simulated until their dissociation.

Supplementary Notes III. ADHESION MODEL WITHOUT UNFOLDING

The following mean-field equations are used to approximate the dynamics of the stretch-dependent state-occupation numbers in the basic adhesion model without molecule unfolding

$$\frac{\partial}{\partial t}N_a = -\beta^+ N_a + \int_{-\infty}^{\infty} \beta^-(h)n_b(h)\,\mathrm{d}h - \gamma^- N_a + \gamma^+\,, \tag{4}$$

$$\frac{\partial}{\partial t}n_b(h) = -v\frac{\partial}{\partial h}n_b(h) - \beta^-(h)n_b(h) + \beta^+(h)n_a(h)\,, \tag{5}$$

where we omitted explicit t-dependence in our notation. Since the unbound molecules in state a are assumed to relax quickly mechanically, their extensions obey a Gaussian distribution. Supplementary Eq. (4) describes the mean total number of unbound molecules. The mean number of bonds $n_b(h)$ evolves according to Supplementary Eq. (5). The drift term accounts for the average relative velocity $v = \langle \dot{s} \rangle$ between the adhesion planes that is due to the tangential force F. An expansion of $n_b(h)$ as

$$n_b(h) = \sum_{j=0}^{\infty} \frac{1}{j!}\, n_{b_j}(h)\, \tilde{v}^j, \tag{6}$$

with $\tilde{v} = v/(k_\beta \sigma_b)$ fulfills the steady state conditions $\frac{\partial}{\partial t}N_a = 0$ and $\frac{\partial}{\partial t}n_b(h) = 0$ if

$$n_{b_0}(h) = n_b^*(h) = \frac{\gamma^+}{\gamma^-}\frac{\beta^+(h)}{\beta^-(h)}\,p_a(h)\,, \quad n_{b_j}(h) = -j\frac{n'_{b_{j-1}}(h)}{\beta^-(h)} \quad \text{for } j > 0\,, \tag{7}$$

where $p_a(h)$ is a Gaussian function with zero mean and variance σ_a^2 and the prime denotes the derivative with respect to the extension h. The first correction $n_{b_1}(h)$ is an odd function, so that its integral vanishes. The first non-vanishing correction is given by the term for $j = 2$. Up to second order, the integrated steady state solution for the mean number of bonds $N_B = N_b$ is given by

$$N_B = \int_{-\infty}^{\infty} n_b(h)\mathrm{d}h = N_B^*\left(1 - \sqrt{\frac{2}{\pi}}\tilde{\ell}_{\mathrm{b}}\mathrm{e}^{\tilde{\ell}_{\mathrm{b}}^2}\left(1 - \sqrt{2\pi}\tilde{\ell}_{\mathrm{b}}\mathrm{e}^{2\tilde{\ell}_{\mathrm{b}}^2}\mathrm{Erfc}\left(\sqrt{2}\tilde{\ell}_{\mathrm{b}}\right)\right)\tilde{v}^2\right) \quad \text{with } N_B^* = \frac{\gamma^+}{\gamma^-}\mathrm{e}^{\bar{\epsilon}_{\mathrm{b}}}\,, \tag{8}$$

where $\tilde{v} = v/(\sigma_b k_\beta)$ and $\tilde{\ell}_{\mathrm{b}} = \ell_{\mathrm{b}}/\sigma_b$ have been used and $\mathrm{Erfc}(x)$ denotes the complementary error function. This first correction $\propto v^2$ is negative since the complementary error function for an argument $x > 0$ is bounded by

$$\mathrm{Erfc}(\sqrt{2}\ell_{\mathrm{b}}) \leq \frac{2}{\sqrt{\pi}}\frac{\mathrm{e}^{-2\tilde{\ell}_{\mathrm{b}}^2}}{\sqrt{2}\tilde{\ell}_{\mathrm{b}} + \sqrt{2\tilde{\ell}_{\mathrm{b}}^2 + 4/\pi}} < \frac{\mathrm{e}^{-2\tilde{\ell}_{\mathrm{b}}^2}}{\sqrt{2\pi}\tilde{\ell}_{\mathrm{b}}}\,. \tag{9}$$

The force-balance equation $F = \int_{-\infty}^{\infty} n_b(h)\kappa_b h\,\mathrm{d}h$ connects the external force with the resulting mean velocity v. The lowest-order contribution ($j = 0$) to the force balance vanishes because $n_{b_0}(h)$ is symmetric. For the first non-vanishing correction, we find

$$\tilde{F} = \frac{F\sigma_b}{k_{\mathrm{B}}T} = N_B^*\mathrm{e}^{\tilde{\ell}_{\mathrm{b}}^2/2}\left(-\frac{2\tilde{\ell}_{\mathrm{b}}}{\sqrt{2\pi}} + \left(1 + \tilde{\ell}_{\mathrm{b}}^2\right)\mathrm{e}^{\tilde{\ell}_{\mathrm{b}}^2/2}\mathrm{Erfc}\left(\frac{\tilde{\ell}_{\mathrm{b}}}{\sqrt{2}}\right)\right)\tilde{v}\,. \tag{10}$$

The bracketed term on the right hand side of Supplementary Eq. (10) is strictly positive. The approximations for $\tilde{\ell}_{\mathrm{b}} \ll 1$ are given in the main text, see Eq. (2). A comparison of simulation averages, the analytical results in Supplementary Eqs. (8) and (10), and the approximations for $\tilde{\ell}_{\mathrm{b}} \ll 1$ are shown in Supplementary Fig. 2.

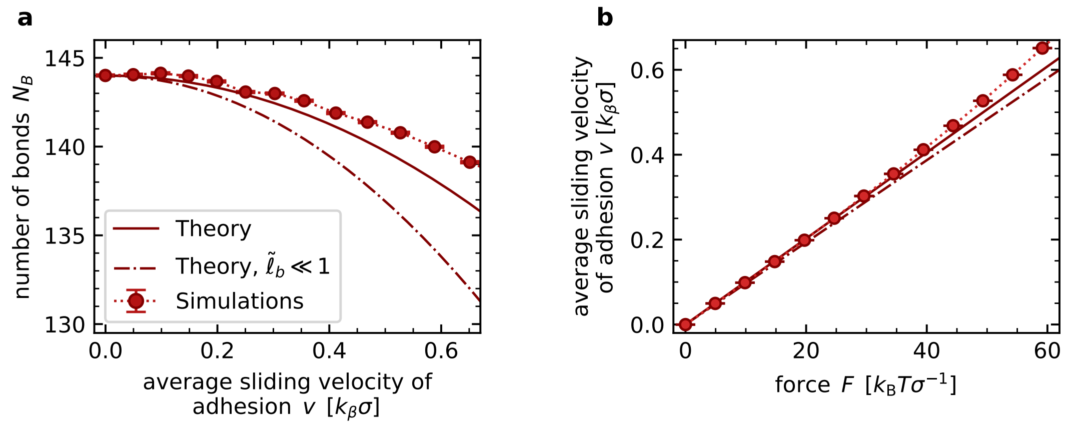

FIG. 2. The average steady-state behavior of basic adhesions without unfolding. Symbols: Simulation results. Solid lines: analytical approximations using an expansion in $\tilde{v}$ up to second order. Dash-dotted lines without markers: Approximation of the expansion for $\tilde{\ell}_\mathrm{b} \ll 1$. a) The analytical approximation slightly underestimates the mean number of bonds in steady state. b) The force-balance condition connects the external force F with the drift v. Parameter values are given in Supplementary Notes I.

Supplementary Notes IV. ADHESION MODEL WITH UNFOLDING

The numbers of unbound molecules obey the following approximate rate equations

$$\frac{\partial}{\partial t} N_a = -\beta^+ N_a + \int_{-\infty}^{\infty} \beta^-(h) n_b(h)\,\mathrm{d}h - \delta_a^+ N_a + \delta_a^- N_{a_\mathrm{u}} - \gamma^- N_a + \gamma^+ \,, \tag{11}$$

$$\frac{\partial}{\partial t} N_{a_\mathrm{u}} = -\beta_\mathrm{u}^+ N_{a_\mathrm{u}} + \int_{-\infty}^{\infty} \beta_\mathrm{u}^-(h) n_{b_\mathrm{u}}(h)\,\mathrm{d}h - \delta_a^- N_{a_\mathrm{u}} + \delta_a^+ N_a \,. \tag{12}$$

The distributions of molecule numbers with extension h experience a drift due to the velocity v of the upper boundary and obey

$$\frac{\partial}{\partial t} n_b(h) = -v\frac{\partial}{\partial h} n_b(h) - \beta^-(h) n_b(h) + \beta^+(h) n_a(h) - \delta_b^+(h) n_b(h) + \delta_b^-(h-\Delta) n_{b_\mathrm{u}}(h-\Delta) \,, \tag{13}$$

$$\frac{\partial}{\partial t} n_{b_\mathrm{u}}(h) = -v\frac{\partial}{\partial h} n_{b_\mathrm{u}}(h) - \beta_\mathrm{u}^-(h) n_{b_\mathrm{u}}(h) + \beta_\mathrm{u}^+(h) n_{a_\mathrm{u}}(h) - \delta_b^-(h) n_{b_\mathrm{u}}(h) + \delta_b^+(h+\Delta) n_b(h+\Delta) \,. \tag{14}$$

The steady-state solution for $v = 0$, i.e., the equilibrium solution, is given by

$$N_a^* = \frac{\gamma^+}{\gamma^-} \,, \qquad N_{a_\mathrm{u}}^* = \frac{\gamma^+}{\gamma^-}\frac{\delta_a^+}{\delta_a^-} \,, \qquad n_b^*(h) = \frac{\gamma^+}{\gamma^-}\frac{\beta^+(h)}{\beta^-(h)} p_a(h) \,, \qquad n_{b_\mathrm{u}}^*(h) = \frac{\gamma^+}{\gamma^-}\frac{\beta_\mathrm{u}^+(h)}{\beta_\mathrm{u}^-(h)} p_{a_\mathrm{u}}(h) \,, \tag{15}$$

where $p_a(h)$ and $p_{a_\mathrm{u}}(h)$ are Gaussian functions with zero mean and variance σ_a^2. Integration yields the total number of bonds in equilibrium as

$$N_B^* = \int_{-\infty}^{\infty} \left[n_b^*(h) + n_{b_\mathrm{u}}^*(h) \right] \mathrm{d}h = \frac{\gamma^+}{\gamma^-} \mathrm{e}^{\tilde{\epsilon}_\mathrm{b}} \left(1 + \mathrm{e}^{-\tilde{\epsilon}_\mathrm{f}} \right) \,. \tag{16}$$

To investigate the case $F > 0$, the state distributions are expanded in powers of the average velocity $\tilde{v}$, see Supplementary Notes III. For the constant term $\propto \tilde{v}^0$, the equilibrium steady state solution is recovered, see Supplementary Eq. (15). For higher orders $\propto \tilde{v}^j$ with $j > 0$, Supplementary Eqs. (13) and (14) are integrated over all stretches. The sum of both integrated equations reads in order j and for the stationary state

$$0 = -\int_{-\infty}^{\infty} \beta^-(h) n_{b_j}(h)\mathrm{d}h - \int_{-\infty}^{\infty} \beta_\mathrm{u}^-(h) n_{b_{\mathrm{u}j}}(h)\mathrm{d}h + \beta^+ N_{a_j} + \beta_\mathrm{u}^+ N_{a_{\mathrm{u}j}} \,. \tag{17}$$

A comparison of Supplementary Eq. (17) with the sum of Supplementary Eqs. (11) and (12) in stationary state

$$0 = -\beta^{+} N_{a_j} - \beta_{\mathrm{u}}^{+} N_{a_{\mathrm{u}j}} + \int_{-\infty}^{\infty} \beta^{-}(h) n_{b_j}(h)\,\mathrm{d}h + \int_{-\infty}^{\infty} \beta_{\mathrm{u}}^{-}(h) n_{b_{\mathrm{u}j}}(h)\,\mathrm{d}h - \gamma^{-} N_{a_j} \tag{18}$$

yields the condition

$$0 = \gamma^{-} N_{a_j}\,. \tag{19}$$

Hence, the connection of the state a with the reservoir enforces $N_{a_j} = 0$ for $j > 0$. In general, the remaining solutions of the stationary equations cannot be found with an exact iterative formula. Instead, a numerical approach is used to find the corrections $N_{a_{\mathrm{u}j}}$, which can then be inserted back into the rate equations to solve for $n_{b_j}(h)$ and $n_{b_{\mathrm{u}j}}(h)$. Up to first order in $\tilde{v}$, the mean number of bonds remains unaffected or increases with the velocity, as demonstrated for different unfolding steps Δ, unfolding energies ϵ_{f}, and optimal binding lengths ℓ_{b} in Fig. 3a,c. The first-order correction vanishes for $\tilde{\Delta} \to 0$, $\tilde{\Delta} \to \infty$ and $\tilde{\epsilon}_{\mathrm{f}} \to \infty$. The second-order correction can be both positive or negative, Fig. 3b,d. Strong self-stabilization is found for $\tilde{\ell}_{\mathrm{b}} \ll 1$.

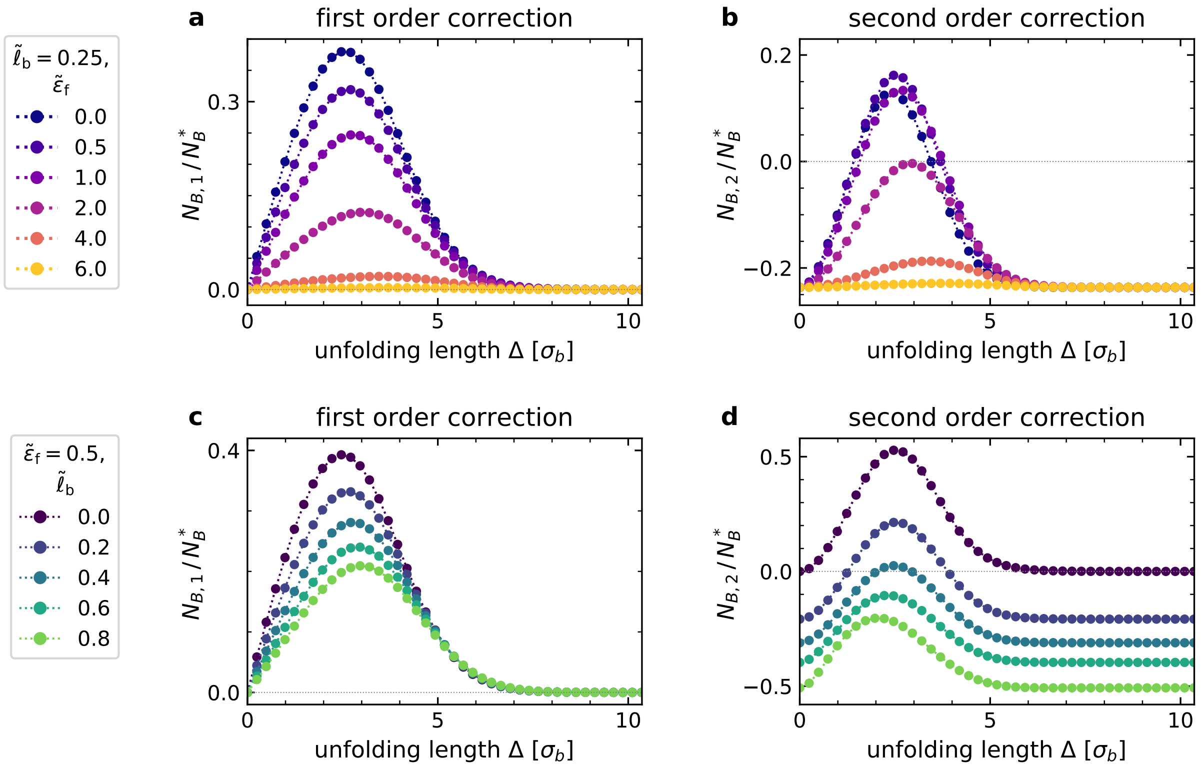

FIG. 3. Numerical results for the expansion of the number of bonds $N_B = N_b + N_{b_{\mathrm{u}}}$ for adhesion clusters with unfolding and state a in exchange with the reservoir. a-b) First and second order correction for different values of unfolding length Δ and unfolding energy ϵ_{f}. c-d) First and second order correction for different values of unfolding length Δ and binding length ℓ_{b}. See Supplementary Notes I for remaining parameters.

The on-average constant sliding velocity v results from continuous cycles of bond binding, unfolding and rupture of individual molecules. This sliding velocity is an indicator for the shift out of equilibrium. The deviation from the equilibrium distribution is illustrated in Fig. 3 and Supplementary Fig. 4. Supplementary Figure 4a shows the ratio of the expected values of the rates $\langle \beta_{(\mathrm{u})} \rangle = \langle \beta_{(\mathrm{u})}^{+}(h) \rangle / \langle \beta_{(\mathrm{u})}^{-}(h) \rangle$ and $\langle \delta_{b,a} \rangle = \langle \delta_{b,a}^{+}(h) \rangle / \langle \delta_{b,a}^{-}(h) \rangle$. In equilibrium, these ratios yield the average occupation number ratio of the involved states, e.g. $\langle \beta \rangle = N_b/N_a$. For non-vanishing forces, the two ratios do not coincide, so that global balance is broken. The deviations indicate an effective flux along the cycle of transitions $\mathrm{a} \rightleftharpoons \mathrm{b} \rightleftharpoons \mathrm{b_u} \rightleftharpoons \mathrm{a_u} \rightleftharpoons \mathrm{a}$, where longer reaction arrows point in the direction of the effective flux. By means of the macroscopic global balance conditions, the number of bonds can be expressed as $N_b = N_a \langle \beta \rangle$ and $N_{b_{\mathrm{u}}} = N_b \langle \delta_b \rangle = N_{a_{\mathrm{u}}} \langle \beta_{\mathrm{u}} \rangle$. Supplementary Fig. 4b shows, that the equality only holds for $\tilde{F} = 0$. For

the total number of bonds $N_B = N_b + N_{b_u}$, the deviations cancel each other, if the average number of unfolded bonds is obtained via the binding process. However, if the value is obtained via the unfolding transition, i.e., $N_{b_u} = N_b\langle\delta_b\rangle$, the equality again only holds at vanishing forces, see Supplementary Fig. 4c.

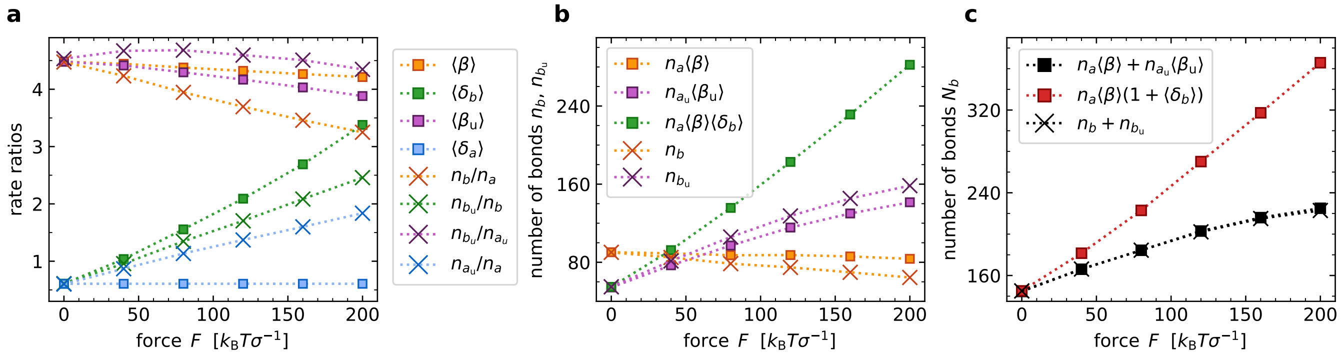

FIG. 4. Steady state simulation results demonstrate the shift out of equilibrium. The global balance conditions only hold at $\tilde{F} = 0$ and are broken for non-vanishing forces. a) Ratios of average state occupation numbers (cross markers) and average rate ratios (square markers, same color). b) Average state occupation numbers of bonds N_b and N_{b_u} (cross markers) and product of neighboring state occupation number and average rate ratio (square markers, same color). c) Average number of bonds calculated via the average state occupation number $N_b + N_{b_u}$ (cross markers) and via the global balance conditions (square markers). Note that the number of unfolded bonds can either be described via binding of unfolded, unbound molecules or via unfolding of bonds in state b. Parameters are given in Supplementary Notes I.

IV.i. Rupture behavior and adhesion cluster lifetimes

Application of a sudden force jump from $F_1 = 0$ to $F_2 > 0$ either leads to a quick dissociation of the adhesion or the adhesion system relaxes to a non-equilibrium steady state. For large adhesion clusters ($N > 20$), the lifetimes of those adhesions that reach the non-equilibrium steady-state usually exceeds the finite simulation time. The fraction of those adhesion clusters that rupture almost immediately, $\Phi_{\rm rupt}$, is shown in Supplementary Fig. 5a as a function

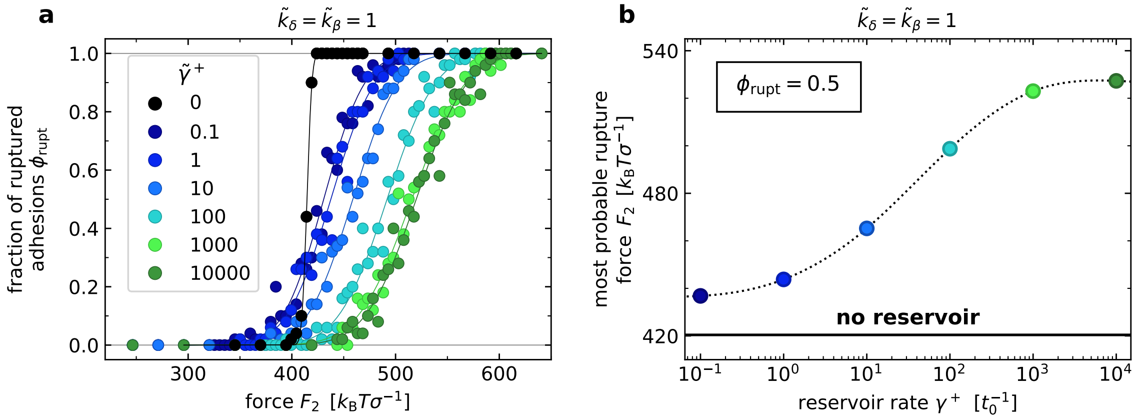

FIG. 5. Fraction of ruptured adhesions after a sudden load-jump from $F_1 = 0$ to F_2 for different reservoir-exchange rates $\gamma^\pm$. The ratio $\gamma = \gamma^+/\gamma^- = 20$ is fixed. Systems were simulated for $5 \cdot 10^5$ reaction steps after the force jump. a) The fraction of ruptured adhesion clusters as a function of the magnitude of the force step. The strength of the reservoir connection, determined by $\gamma^\pm$, is varied while the ratio $\gamma = \gamma^+/\gamma^-$ is held constant. For comparison, the fraction of ruptured adhesion clusters in simulations without reservoir connection is shown with black bullets. A reservoir connection implies here self-stabilization. Due to the large system size ($N^* \approx 176$), most systems that did not rupture initially after the load jump remained stable for the rest of the simulation time. Lines show fits to error functions. b) Force F_2 at which rupture is most likely for different reservoir rate values γ^+. Values are extracted from the fit curves in a). The black horizontal line shows the most probable rupture force for $\gamma^+ = 0$. See Supplementary Notes I for remaining parameters.

of F_2 for different reservoir rates γ^+. Without reservoir connection, when no self-stabilization occurs, the fraction of ruptured adhesion clusters sharply increases at a threshold force. Self-stabilization broadens the curves and increases the typical forces at which rupture occurs. The numerical results are well-fitted by a shifted and scaled error function.

The fraction of ruptured adhesion clusters after sufficiently long simulation times represents the cumulative probability distribution for cluster dissociation after a sudden force application. Therefore, the force value F_2 at which $\Phi_{\mathrm{rupt}}(F_2) = 0.5$ corresponds to the highest rupture probability, see Supplementary Fig. 5b. The most probable rupture force increases with increasing reservoir rate γ^+.

Supplementary Figure 6 shows adhesion lifetimes, defined as the time until first complete dissociation of all bonds in an adhesion after application of a force jump. Small adhesion clusters ($N^* \approx 10$) with different values for the reservoir rates $\gamma^\pm$ are studied. The small molecule-numbers allow a direct measurement of the lifetimes by simulating the clusters until the last bond dissociates. For an adhesion system without molecule unfolding, realized by setting $k_\delta = 0$, the lifetime decreases monotonically with increasing force for any value of $\gamma^\pm$, see Supplementary Fig. 6a. The equilibrium lifetime is largest for adhesion clusters without reservoir connection, realized by $\gamma^\pm = 0$. Supplementary Figs 6b,c display lifetimes of adhesion models with unfolding molecules. The lifetime of adhesions without molecule exchange with a reservoir decrease monotonically with increasing force (black markers). However, when the system is coupled to a molecule reservoir, so that the self-stabilization mechanism takes effect, the adhesion lifetime curves have a maximum at non-zero, finite forces. A substantial lifetime increase through self-stabilization is realized when both the reservoir rates and the intrinsic binding rate k_β are large compared to the unfolding rate k_δ.

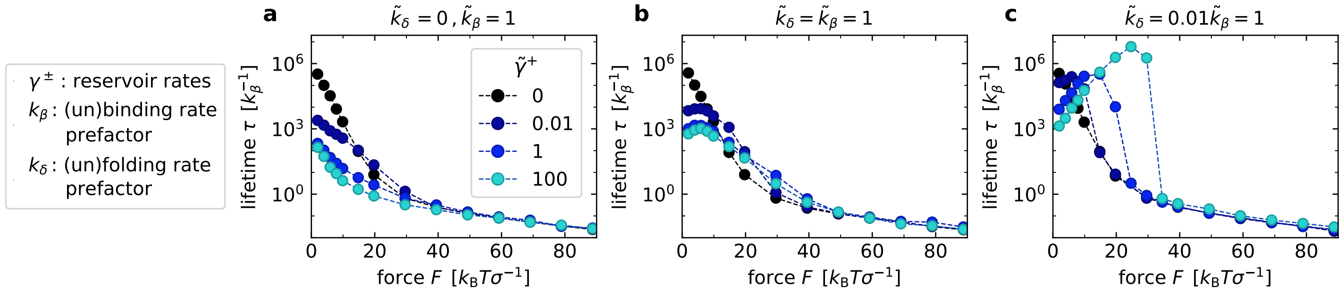

FIG. 6. Lifetimes of small adhesions ($N^* \approx 10$) for different values of the rate prefactors k_β and k_δ and the reservoir-exchange rates $\gamma^\pm$. The ratio $\gamma = \gamma^+/\gamma^- = 1$ is fixed. a) $k_\delta = 0$, no unfolding b) $k_\delta = k_\beta$ c) $k_\delta = 0.01 k_\beta$. Only the lifetime results for the adhesion model IV, which exhibits self-stabilization, display a maximum at finite, non-vanishing forces. The lifetime of the adhesions is substantially increased for intermediate forces if γ^+ and k_β are larger than k_δ, such that molecules coming from the reservoir establish new bonds frequently. See Supplementary Notes I for remaining parameters.

IV.ii. Special case: no unfolding in the unbound state a

To investigate how cyclic fluxes along the state network $a - b - b_{\mathrm{u}} - a_{\mathrm{u}} - a$ affect self-stabilization, we set $\delta_a^\pm = 0$. Thereby, the cycle in the single-molecule transition-diagram is broken. Physically, this modification means that unfolding is only allowed when the molecule is bound between both planes. It should be emphasized that this model variant still allows the emergence of cyclic fluxes in the high-dimensional continuous state space spanned by the extensions of the molecules.

In Supplementary Fig. 7, results from the new model variant are compared with results from model IV defined in the main text. For $\delta_a^\pm = 0$, the increase of the adhesion molecule number with force is significantly stronger than for the model IV with cyclic flux. Thus, self-stabilization is enhanced in the absence of cyclic flux, see Supplementary Figs. 7a,b. The relative velocity of the two planes bounding the adhesion is reduced accordingly, see Supplementary Fig. 7c. The enhanced self-stabilization can be attributed to a stronger accumulation of molecules in the state a_{u}, from which molecules can only escape via state b_{u} in this model variant. Note that the force value at which first rupture events are observed does not increase greatly, see black, vertical lines in Supplementary Fig. 7a. A comparison with the inset in Supplementary Fig. 7b shows, that the force per bond, F/N_B, is strongly reduced because of the increased number of bonds N_B.

The absence of cyclic fluxes is further illustrated in Supplementary Figs. 8 and 9. The results in Supplementary Fig. 8 demonstrate that fluxes occur between the states $b_{\mathrm{u}}(h_{\mathrm{u}})$ and $a_{\mathrm{u}}(h_{\mathrm{u}})$. The distributions of both states are shown in Supplementary Fig. 8a. In steady state, the stretch change per transition due to molecule binding vanishes on average, while the average stretch change due to bond rupture is positive for $\tilde{F} > 0$. Similarly, the average shift of

the adhesion planes is positive, see Supplementary Fig. 8b. Supplementary Fig. 8c shows the effective particle flux between b_{u} and a_{u} as a function of the stretch value h_{u}. At negative and small positive stretches, there is an effective particle flux from a_{u} to b_{u} for $\tilde{F} > 0$. At large positive stretches, rupture dominates, so that there is an effective flux from b_{u} to a_{u} for positive forces. Supplementary Fig. 9 shows that the global balance conditions do not only hold at $\tilde{F} = 0$, but also for non-vanishing forces. The average rate ratios of neighbouring states agree with the ratio of the average state occupation numbers.

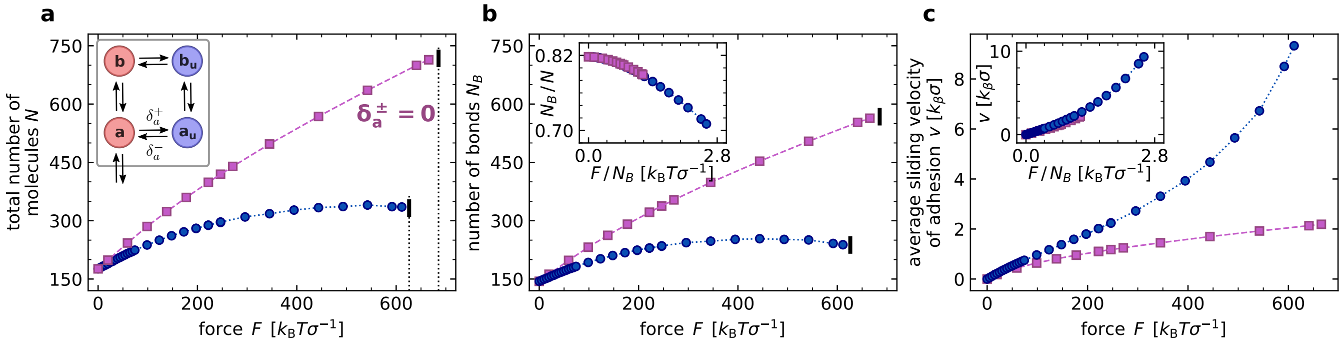

FIG. 7. Steady state results of adhesion model IV (compare Fig. 2 in the main text) and adhesion clusters with $\delta_a^{\pm} = 0$. When unbound unfolding and refolding is inhibited, the self-stabilization effect is more pronounced. See Supplementary Notes I for parameters.

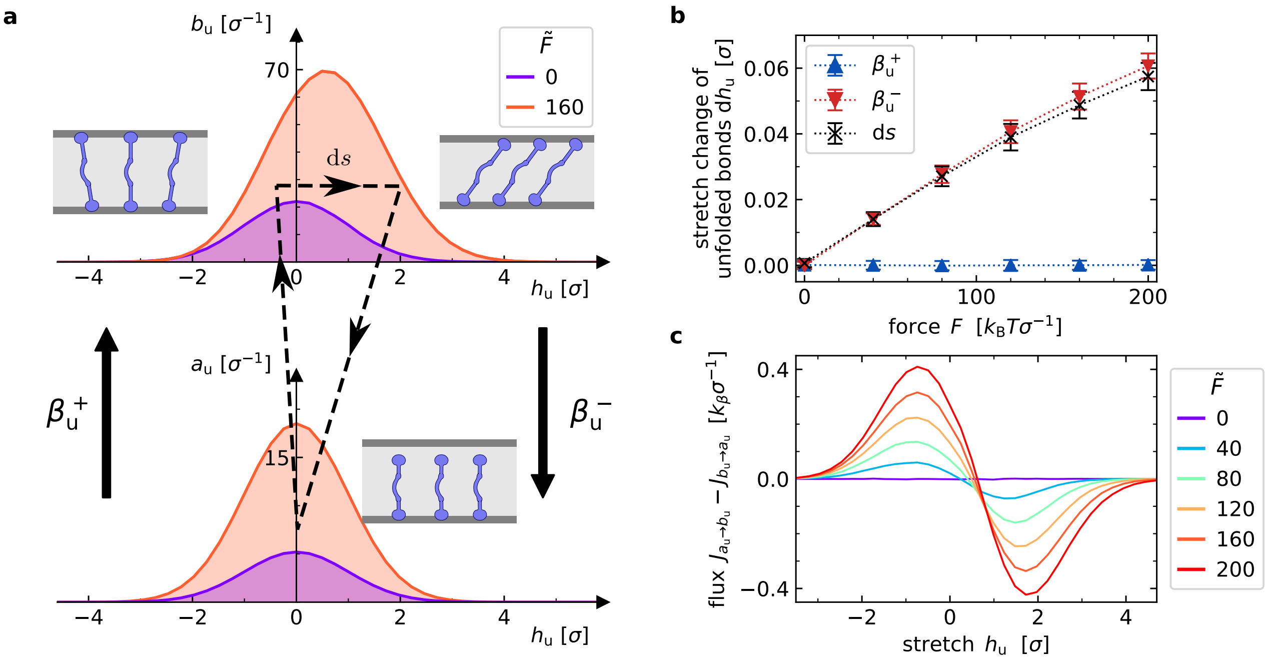

FIG. 8. Steady state fluxes between unfolded bonds and unfolded, unbound molecules for the model variant where $\delta_a^{\pm} = 0$. Compare Fig. 3 of the main text. a) Average stretch distribution of unfolded molecules $b_{\mathrm{u}}(h)$ and $a_{\mathrm{u}}(h)$ at vanishing and finite forces. Unfolded bonds exhibit an on average positively skewed distribution under force in steady state. b) The average stretch change per transition for unfolded bonds due to binding events (β_{u}^{+}), bond rupture (β_{u}^{-}) and the average shift of the boundary ds as a function of force. Binding occurs symmetrically around $h = 0$. Error bars indicate the sample standard deviation. c) Flux balance distribution for binding and unbinding of unfolded molecules. At vanishing forces, all fluxes are balanced. A net particle flux into state b_{u} at negative stretches and a net particle flux out of state b_{u} at positive stretches is found for $\tilde{F} > 0$. Fluxes are obtained by binning the transition rates that are chosen in steady state simulation trajectories with a bin width $0.25\sigma_b$. See Supplementary Notes I for parameters.

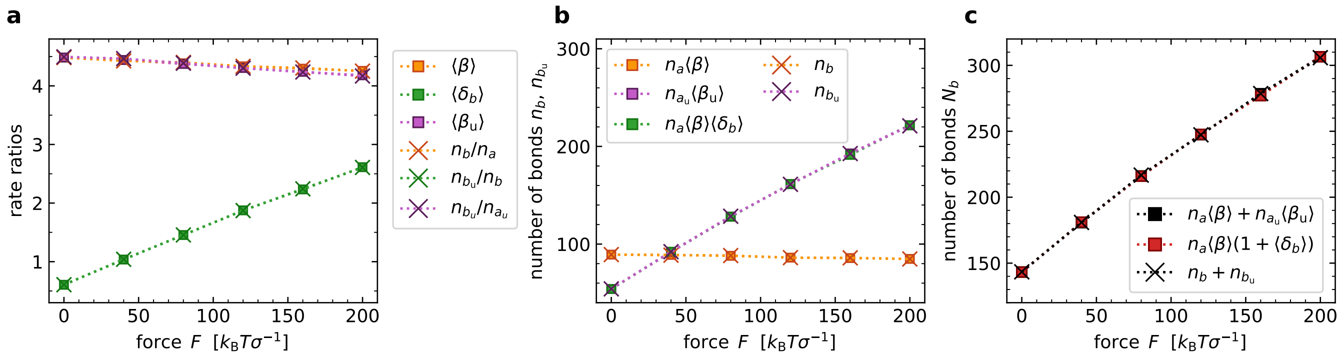

FIG. 9. Steady state simulation demonstrate that global balance holds for $\delta_a^{\pm} = 0$. a) Ratios of average state occupation numbers (cross markers) and average rate ratios (square markers, same color). b) Average state occupation numbers of bonds N_b and N_{b_u} (cross markers) and product of neighboring state occupation number and average rate ratio (square markers, same color). c) Average number of bonds calculated via the average state occupation number $N_b + N_{b_u}$ (cross markers) and via the global balance conditions (square markers). Note that the number of unfolded bonds can either be described via binding of unfolded, unbound molecules or via unfolding of bonds in state b. See Supplementary Notes I for parameters.

Supplementary Notes V. ADHESION MODEL WITH UNFOLDING AND CATCH BONDS

In the models considered so far, a slip-bond behavior is assumed, i.e., the increase of the single-bond rupture rate with the applied extension h is monotonic, see sec. IV B. Thus, so far, single-bond lifetimes decreased when tension increased. Bonds that become longer-lived when tension increases are called catch bonds [23]. A number of molecular bonds in cellular adhesions have been described as catch-slip bonds [24–27]. These interactions behave like a catch bond up to some force threshold while the slip-bond behavior takes effect at higher forces. To further test the generality of the self-stabilization mechanism, simulations of different adhesion models are performed where the bonds behave like pure catch bonds. The binding and unbinding rate of folded bonds are changed to

$$\beta^+(h)\frac{n_a(h)}{N_a} = \frac{k_\beta}{\sqrt{2\pi}\sigma_b}\exp\left(-\frac{\left(|h|+\ell_\mathrm{b}\right)^2}{2\sigma_b^2}+\frac{\epsilon_\mathrm{b}}{k_\mathrm{B}T}\right), \qquad \beta^-(h) = k_\beta\exp\left(\frac{-2|h|\ell_\mathrm{b}-\ell_\mathrm{b}^2}{2\sigma_b^2}\right). \tag{20}$$

The rates for unfolded bonds are defined analogously with the extension h_u. Note that the difference to the slip bond rates given in Methods A-B lies only in one sign in each exponential function. Thus, detailed balance still holds in equilibrium at $\tilde{F} = 0$. The steady-state simulation results are shown in Supplementary Fig. 10. Again, only for model IV with a combination of unfolding and association of new molecules from the reservoir, a pronounced increase in the number of bonds is observed for small forces, see Supplementary Fig. 10b. Supplementary Figure 10d shows that the self-stabilization is caused by an accumulation of unbound molecules. Beyond this regime, at high forces, the pure catch-bond dynamics leads to a separation of the bond distribution into two subpopulations. Firstly, one has few, rather static molecules that carry most of the tension. Therefore, these molecules have a large extension and long lifetime. Secondly, a large number molecules form transient bonds with low, symmetrically distributed extensions. As a result, the cluster stops moving, as can be seen in the velocity-force curve in Supplementary Fig. 10c for high forces.

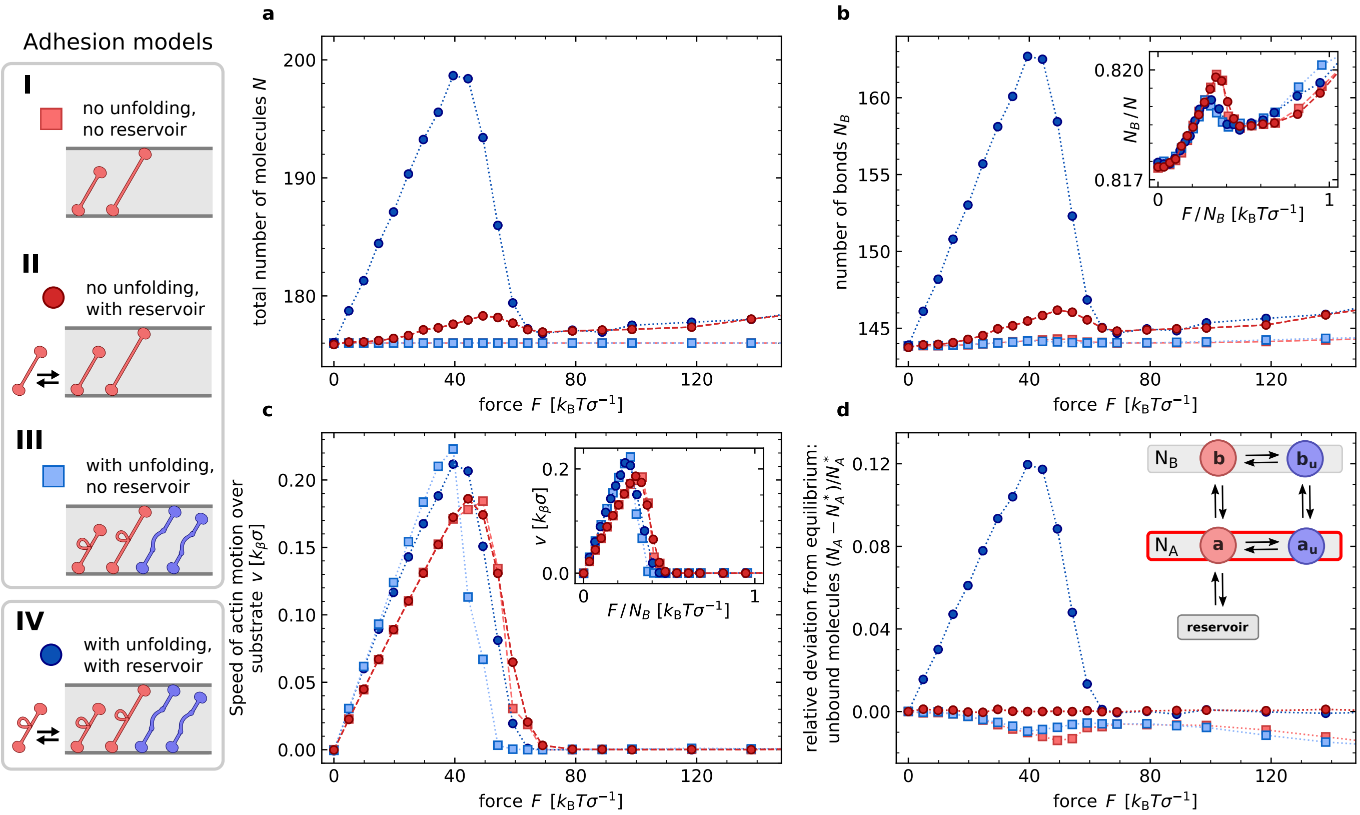

FIG. 10. Simulation results for adhesion models in which single molecules follow a catch-bond behaviour. a) Averaged total number of molecules N in steady state. b) Averaged number of bonds N_B in steady state. Adhesion clusters with a reservoir connection (dark red and dark blue) show a regime, where the mean number of bonds increases with force. The self-stabilizing mechanism is particularly pronounced for the model IV (dark blue) in which molecules can unfold and are in exchange with a reservoir. c) The continuous binding and rupture leads to an on average constant relative shift of the boundaries per time. Adhesion models with bond unfolding show an increased velocity. d) The relative deviation of the average number of unbound molecules N_A from equilibrium shows that the self-stabilization mechanism is connected to an increased accumulation of adhesion molecules in unbound states (dark blue). The parameter values are given in Supplementary Notes I.

Supplementary Notes VI. ADHESION MODEL WITH UNFOLDING AND CROSS-LINKING

In the following an extended adhesion model is considered that also contains the effect of adaptor protein binding. Supplementary Figure 11a shows the corresponding single-molecule state-transition diagram with linking rates $\lambda^{\pm}$. Linking the load-bearing molecules with adaptor proteins to the adhesion prevents a dissociation of the molecules from the adhesion. Thereby, the linking generates states that are not directly connected to the molecule reservoir.

For an approximate analytical description of the system, the set of equations from Supplementary Notes IV is extended to account for the linked states. The unbound states obey

$$\frac{\partial}{\partial t} N_a = -\beta^+ N_a + \int_{-\infty}^{\infty} \beta^-(h) n_b(h)\,\mathrm{d}h - \delta_a^+ N_a + \delta_a^- N_{a_\mathrm{u}} - \gamma^- N_a + \gamma^+ \,, \tag{21}$$

$$\frac{\partial}{\partial t} N_{a_\mathrm{u}} = -\beta_\mathrm{u}^+ N_{a_\mathrm{u}} + \int_{-\infty}^{\infty} \beta_\mathrm{u}^-(h) n_{b_\mathrm{u}}(h)\,\mathrm{d}h - \delta_a^- N_{a_\mathrm{u}} + \delta_a^+ N_a - \gamma^- N_{a_\mathrm{u}} + \gamma^+ \frac{\delta_a^+}{\delta_a^-} - \lambda^+ N_{a_\mathrm{u}} + \lambda^- N_{a_{\mathrm{u},1}} \,, \tag{22}$$

$$\frac{\partial}{\partial t} N_{a_{\mathrm{u},1}} = -\beta_\mathrm{u}^+ N_{a_{\mathrm{u},1}} + \int_{-\infty}^{\infty} \beta_\mathrm{u}^-(h) n_{b_{\mathrm{u},1}}(h)\,\mathrm{d}h - \lambda^- N_{a_{\mathrm{u},1}} + \lambda^+ N_{a_\mathrm{u}} \,. \tag{23}$$

The equations determining the evolution of the extension-dependent state distributions read

$$\frac{\partial}{\partial t} n_b(h) = -v \frac{\partial}{\partial h} n_b(h) - \beta^-(h) n_b(h) + \beta^+(h) n_a(h) - \delta_b^+(h) n_b(h) + \delta_b^-(h-\Delta) n_{b_\mathrm{u}}(h-\Delta)\,, \tag{24}$$

$$\begin{aligned}\frac{\partial}{\partial t} n_{b_\mathrm{u}}(h) = -v \frac{\partial}{\partial h} n_{b_\mathrm{u}}(h) - \beta_\mathrm{u}^-(h) n_{b_\mathrm{u}}(h) + \beta_\mathrm{u}^+(h) n_{a_\mathrm{u}}(h) - \delta_b^-(h) n_{b_\mathrm{u}}(h) + \delta_b^+(h+\Delta) n_b(h+\Delta)\\ - \lambda^+ n_{b_\mathrm{u}}(h) + \lambda^- n_{b_{\mathrm{u},1}}(h)\,,\end{aligned} \tag{25}$$

$$\frac{\partial}{\partial t} n_{b_{\mathrm{u},1}}(h) = -v \frac{\partial}{\partial h} n_{b_{\mathrm{u},1}}(h) - \beta_\mathrm{u}^-(h) n_{b_{\mathrm{u},1}}(h) + \beta_u^+(h) n_{a_{\mathrm{u},1}}(h) - \lambda^- n_{b_{\mathrm{u},1}}(h) + \lambda^+ n_{b_u}(h)\,. \tag{26}$$

The steady-state results for the number of molecules in state a, b, b_u, a_u are given in Supplementary Eq. (15). The additional linked states fulfill

$$n^*_{b_{\mathrm{u},1}}(h) = \frac{\lambda+}{\lambda^-} n^*_{b_\mathrm{u}}(h)\,, \quad N^*_{a_{\mathrm{u},1}} = \frac{\lambda+}{\lambda^-} N^*_{a_\mathrm{u}}\,. \tag{27}$$

As before, an expansion of the distributions for small speeds $|\tilde{v}|$ yields the first corrections to the equilibrium distribution. Supplementary Figure 11b shows a comparison between steady-state results obtained in simulations and in the analytical approximation. Supplementary Figure 11c and d show simulation results for different linking rates λ.

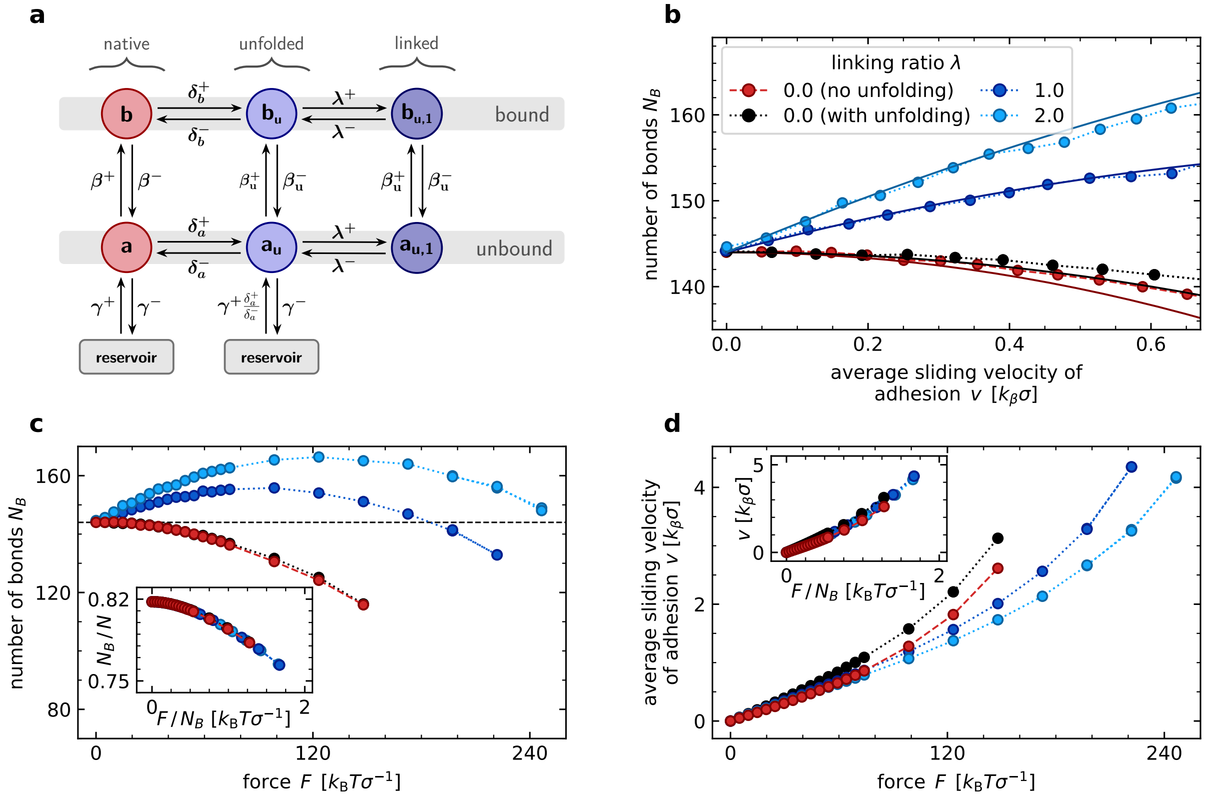

FIG. 11. Model for adhesion clusters with self-stabilization realized by linking of auxiliary molecules. a) Single-molecule state transition diagram. Unfolded molecules can establish an additional link to the adhesion that prevents one-step dissociation of the molecule from the adhesion. b) Comparison between simulation results (markers connected by dashed lines) and the expansion of bond states up to second order in the speed $|\tilde{v}|$ (solid lines) for different linking ratios λ (black and blue markers). Results for an adhesion model without the unfolding transition are shown for comparison (red markers). c) Only adhesions with a positive linking ratio show a self-stabilization regime where the number of bonds increases with force. d) Relative motion of the upper plane and the adhesion. Insets: The fraction of bonds and the velocity as a function of force per bond show the same behavior for all systems. Parameter values are given in Supplementary Notes I.

In this extended model, both unbound states a and a_u are in contact with a particle reservoir. For all orders $\propto \tilde{v}^j$ with $j > 0$, Supplementary Eqs. (24), (26) and (25) are integrated over all stretches. The sum of the three integrated

equations reads in order j and for the stationary state

$$0 = -\int_{-\infty}^{\infty} \beta^-(h) n_{b_j}(h) \mathrm{d}h - \int_{-\infty}^{\infty} \beta_{\mathrm{u}}^-(h) n_{b_{\mathrm{u}j}}(h) \mathrm{d}h - \int_{-\infty}^{\infty} \beta_{\mathrm{u}}^-(h) n_{b_{\mathrm{u},1j}}(h) \mathrm{d}h + \beta^+ N_{a_j} + \beta_{\mathrm{u}}^+ N_{a_{\mathrm{u}j}} + \beta_{\mathrm{u}}^+ N_{a_{\mathrm{u},1j}} \,. \tag{28}$$

A comparison of Supplementary Eq. (28) with the sum of Supplementary Eqs. (21), (22) and (23) in stationary state yields the condition

$$0 = \gamma^-(N_{a_j} + N_{a_{\mathrm{u}j}}) \,. \tag{29}$$

Hence, the reservoir connections enforce $N_{a_j} + N_{a_{\mathrm{u}j}} = 0$ for $j > 0$. In the special case $\lambda^\pm = 0$, unbound molecules can thus not be accumulated. Numerical analysis shows that the first order correction for the change of bound molecules with $\tilde{v}$ can be positive or negative, but the negative second order correction is two orders of magnitude larger and hence dominates already at low velocities, see Supplementary Fig. 12.

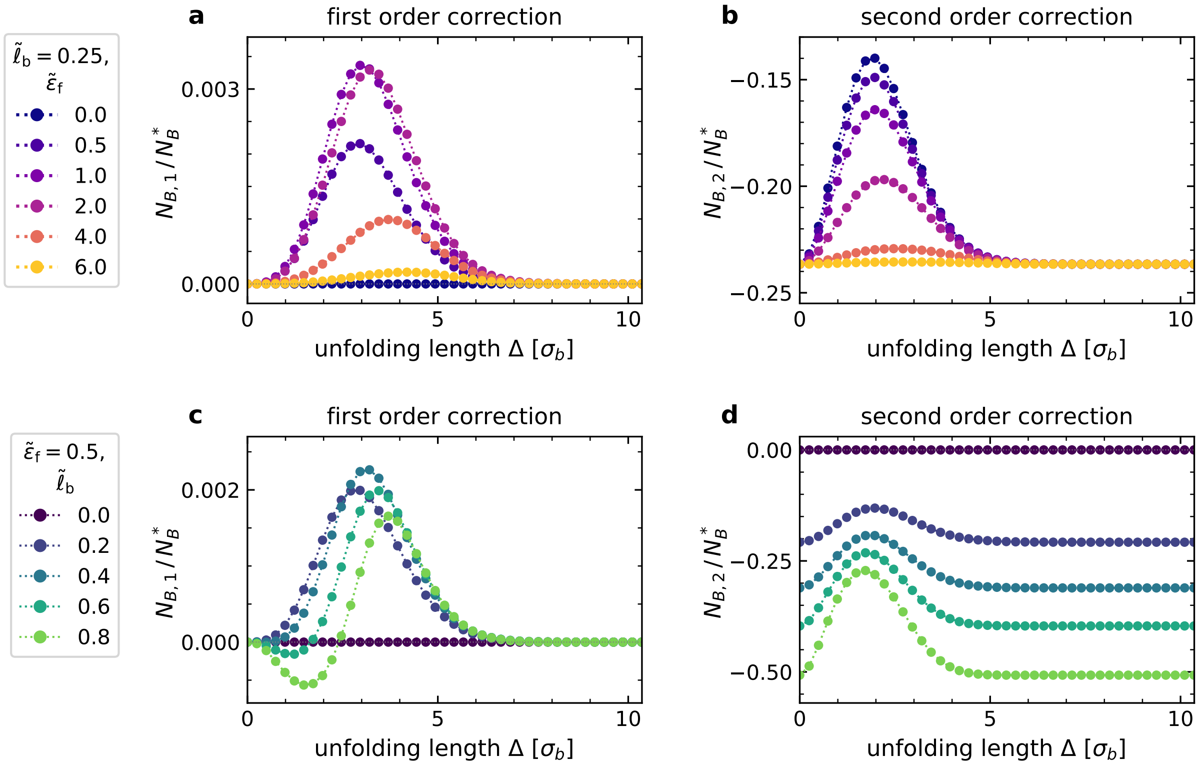

FIG. 12. Numerical results for the expansion of the number of bonds $N_B = N_b + N_{b_\mathrm{u}}$ for adhesion clusters no linking $\lambda = 0$, but both a and a_u in exchange with the reservoir. a,b) First and second order correction for different values of unfolding length Δ and unfolding energy ϵ_f. c,d) First and second order correction for different values of unfolding length Δ and binding length ℓ_b. See Supplementary Notes I for remaining parameters.

Supplementary Notes VII. EXPERIMENTAL MODEL TESTS FOR FOCAL ADHESIONS

The model is inspired by observations made for cell-matrix adhesions that are based on integrins and adaptor proteins such as talin and vinculin. Therefore, the question naturally arises, if the self-stabilization mechanism predicted by the model can be experimentally studied. Clearly, the biological complexity of focal adhesions makes a direct comparison of models and experimental data a challenging task. The self-stabilization mechanism may be realized in various ways and may also be masked by the complex regulatory system at cell adhesions. Experiments on reduced *in vitro* systems, such as micro-clusters of integrin, talin and vinculin molecules, which bind to actin gels [28], may facilitate testing of the suggested self-stabilization mechanism. In the following, a few points of comparison are suggested.

The self-stabilization mechanism is based on the idea that a net influx of adhesion molecules is achieved by a shift of state occupations under load. Molecules can be accumulated because one or more states are not directly in contact with a reservoir held at constant chemical potential and can therefore adapt their occupation numbers. As a result, the molecule cluster size and the lifetime can increase under loading. A targeted measurement of the exchange rates of different conformational states of talin or talin-vinculin complexes between the adhesion site and the surrounding cytosol could help to investigate the occurrence of the self-stabilization mechanism further.

The model suggests that adhesion growth can be observed even though secondary cross-links, such as vinculin, do not transmit forces. Experiments with vinculin mutants, where vinculin cannot transmit mechanical load may provide further inside to the importance of protein recruitment to unfolded talin domains for adhesion growth and strengthening, compare also [29].

It is further expected that the shearing geometry favours self-stabilization. A low surface separation allows a broad distribution of bond stretches h and h_{u} and a sliding motion of the adhesion. In a comparable system under normal, pulling forces, the possible range of binding stretches would be reduced with increasing surface separation. Therefore, the model predicts that self-stabilization is less effective under pulling forces.

A summary of experimental tests that could support the suggested self-stabilization mechanism is given in Table 12.

Test	Hypothesis
- Measuring molecule exchange rates of folded talin between adhesion and cytosol	- Frequent exchange of folded talin favours self-stabilization.
- Fluorescent labelling of talin-vinculin complexes	- Adhesion growth coincides with accumulation of talin-vinculin complexes.
- Impeding vinculin-actin binding or force transfer along vinculin	- Adhesion growth still coincides with accumulation of talin-vinculin complexes.
- Comparing adhesion sites under pulling forces and shearing forces	- The shearing geometry favours self-stabilization.

TABLE 12. Suggestions for testing aspects of the self-stabilization mechanism in experiments.

[1] Kong, D., Ji, B. & Dai, L. Stability of adhesion clusters and cell reorientation under lateral cyclic tension. *Biophys. J.* **95**, 4034–4044 (2008).

[2] Roca-Cusachs, P., Iskratsch, T. & Sheetz, M. P. Finding the weakest link – exploring integrin-mediated mechanical molecular pathways. *J. Cell Sci.* **125**, 3025–3038 (2012).

[3] Erdmann, T. & Schwarz, U. S. Bistability of cell-matrix adhesions resulting from nonlinear receptor-ligand dynamics. *Biophys. J.* **91**, 60–62 (2006).

[4] Schwarz, U. S., Erdmann, T. & Bischofs, I. B. Focal adhesions as mechanosensors: the two-spring model. *BioSystems* **83**, 225–232 (2006).

[5] Qian, J. & Gao, H. Soft matrices suppress cooperative behaviors among receptor-ligand bonds in cell adhesion. *PLoS ONE* **5**, e12342 (2010).

[6] Fenz, S. F. *et al.* Membrane fluctuations mediate lateral interaction between cadherin bonds. *Nat. Phys.* **13**, 906–913 (2017).

[7] Gupton, S. L. & Waterman-Storer, C. M. Spatiotemporal feedback between actomyosin and focal-adhesion systems optimizes rapid cell migration. *Cell* **125**, 1361–1374 (2006).

[8] Bihr, T., Seifert, U. & Smith, A. S. Nucleation of ligand-receptor domains in membrane adhesion. *Phys. Rev. Lett.* **109**, 258101 (2012).

[9] Bihr, T., Seifert, U. & Smith, A. S. Multiscale approaches to protein-mediated interactions between membranes - Relating microscopic and macroscopic dynamics in radially growing adhesions. *New J. Phys.* **17**, 083016 (2015).

[10] Yao, M. *et al.* The mechanical response of talin. *Nat. Commun.* **7**, 11966 (2016).

[11] Yao, M. *et al.* Mechanical activation of vinculin binding to talin locks talin in an unfolded conformation. *Sci. Rep.* **4**, 4610 (2014).

[12] Tapia-Rojo, R., Alonso-Caballero, A. & Fernandez, J. M. Direct observation of a coil-to-helix contraction triggered by vinculin binding to talin. *Sci. Adv.* **6**, eaaz4707 (2020).

[13] Hirata, H., Tatsumi, H., Lim, C. T. & Sokabe, M. Force-dependent vinculin binding to talin in live cells: a crucial step in anchoring the actin cytoskeleton to focal adhesions. *Am. J. Physiol. Cell Physiol.* **306**, C607–C620 (2014).

[14] Ciobanasu, C., Faivre, B. & Le Clainche, C. Actomyosin-dependent formation of the mechanosensitive talin-vinculin complex reinforces actin anchoring. *Nat. Commun.* **5**, 3095 (2014).

[15] Kluger, C. *et al.* Different vinculin binding sites use the same mechanism to regulate directional force transduction. *Biophys. J.* **118**, 1344–1356 (2020).

[16] Moreno-Layseca, P., Icha, J., Hamidi, H. & Ivaska, J. Integrin trafficking in cells and tissues. *Nat. Cell Biol.* **21**, 122–132 (2019).

[17] Kechagia, J. Z., Ivaska, J. & Roca-Cusachs, P. Integrins as biomechanical sensors of the microenvironment. *Nat. Rev. Mol. Cell Biol.* **20**, 457–473 (2019).

[18] Klapholz, B. & Brown, N. H. Talin — The master of integrin adhesions. *J. Cell Sci.* **130**, 2435–2446 (2017).

[19] Lele, T. P., Thodeti, C. K., Pendse, J. & Ingber, D. E. Investigating complexity of protein-protein interactions in focal adhesions. *Biochem. Biophys. Res. Commun.* **369**, 929–934 (2008).

[20] Stutchbury, B., Atherton, P., Tsang, R., Wang, D. Y. & Ballestrem, C. Distinct focal adhesion protein modules control different aspects of mechanotransduction. *J. Cell Sci.* **130**, 1612–1624 (2017).

[21] Gillespie, D. T. A general method for numerically simulating the stochastic time evolution of coupled chemical reactions. *J. Comput. Phys.* **22**, 403–434 (1976).

[22] Gillespie, D. T. Exact stochastic simulation of coupled chemical reactions. *J. Phys. Chem.* **81**, 2340–2361 (1977).

[23] Wang, Y., Yan, J. & Goult, B. T. Force-dependent binding constants. *Biochemistry* **58**, 4696–4709 (2019).

[24] Marshall, B. T. *et al.* Direct observation of catch bonds involving cell-adhesion molecules. *Nature* **423**, 190–193 (2003).

[25] Kong, F., García, A. J., Mould, A. P., Humphries, M. J. & Zhu, C. Demonstration of catch bonds between an integrin and its ligand. *J. Cell Biol.* **185**, 1275–1284 (2009).

[26] Chen, Y., Lee, H., Tong, H., Schwartz, M. & Zhu, C. Force regulated conformational change of integrin $\alpha_V\beta_3$. *Matrix Biol.* **60-61**, 70–85 (2017).

[27] Manibog, K., Li, H., Rakshit, S. & Sivasankar, S. Resolving the molecular mechanism of cadherin catch bond formation. *Nat. Commun.* **5**, 1–11 (2014).

[28] Sackmann, E. & Smith, A. S. Physics of cell adhesion: Some lessons from cell-mimetic systems. *Soft Matter* **10**, 1644–1659 (2014).

[29] Dumbauld, D. W. *et al.* How vinculin regulates force transmission. *Proc. Nat. Acad. Sci. U. S. A.* **110**, 9788–9793 (2013).
