# Supplementary material for: Generic self-stabilization mechanism for biomolecular adhesions under load
Source: Nat Commun. 2022 Apr 22;13:2197. doi: 10.1038/s41467-022-29823-2 (PMC9033785; doi:10.1038/s41467-022-29823-2)
Supplement: Supplementary file 2 — Description of Additional Supplementary Files [file 41467_2022_29823_MOESM2_ESM.pdf]

Description of Additional Supplementary Files

File Name: Supplementary Movie 1
Description: Basic adhesion model – Shear force promotes dissociation

File Name: Supplementary Movie 2
Description: Self-stabilization mechanism – Shear force drives adhesion growth
